# Graphical Approach of the Interplay of Eco‐Evolutionary Dynamics and Coexistence

**DOI:** 10.1111/ele.70470

**Published:** 2026-11-13

**Authors:** Nicolas Loeuille, Rudolf P. Rohr

**Affiliations:** ^1^ Institute of Ecology and Environmental Sciences, IEES, Sorbonne Université, UPEC, CNRS, IRD, INRA Paris France; ^2^ Department of Biology — Ecology and Evolution University of Fribourg Fribourg Switzerland

**Keywords:** adaptive dynamics, species resistance, structural stability

## Abstract

Given the evidence that evolution can affect ecological dynamics, a key question is how such eco‐evolutionary dynamics change the coexistence of species and biodiversity in general. We propose a graphical approach that tackles coexistence and evolution in the same parameter space, allowing direct links between ecological and evolutionary perspectives. We use the approach to highlight conditions under which evolution favours or harms coexistence. Even when focusing on the fate of the evolving species, evolution often leads to greater vulnerability. The graphical approach is based on structural stability approaches, and it can be applied to all interaction types and covers variations in trade‐off structures. Using this flexibility, we highlight how evolution affects positively or negatively coexistence, depending on these two components. Finally, we demonstrate how this approach can be applied to empirical cases. We thereby highlight the critical ingredients needed to inform the graphical approach, its potential use for proposing testable scenarios, but also clarify its limits.

## Graphical abstract

We introduce a graphical tool that bridges eco‐evolutionary dynamics and coexistence theory. (A) Coexistence is quantified by two complementary metrics. At the community level, the angle Ω measures the overall coexistence potential. At the species level, the angles ηᵢ measure each species' resistance to exclusion. Both are drawn directly from species' intrinsic growth rates and interaction coefficients. (B) Evolutionary changes in these parameters, here a decrease in r₁ traded against weaker intraspecific competition (α₁₁), appear as arrows (red, blue) on this geometry. This shows directly how evolution alters Ω and ηᵢ.

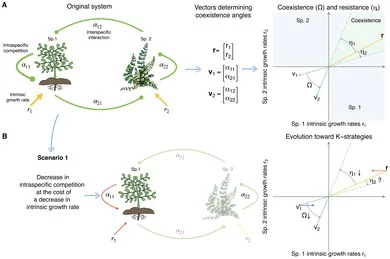

## 1 Introduction

Traditionally, the maintenance of species diversity within ecological communities has been explained through a combination of abiotic constraints (environmental filtering, Kraft et al. (2015)) and biotic interactions among local species (e.g., competitive exclusion (Thorn et al. 2016), intransitive competition (Vandermeer and Perfecto 2023), vertical food‐chain assembly (Hairston et al. 1960; Oksanen et al. 1981)), two processes that are often difficult to disentangle (Cadotte and Tucker 2017). While these ecological components provide a fundamental basis for coexistence, recent works have stressed how evolution can also directly affect ecological dynamics (Hairston et al. 2005; Carroll et al. 2014). Such evolutionary dynamics alter local interactions and environmental responses, thereby reshaping coexistence conditions. Understanding how eco‐evolutionary dynamics influence community assembly has therefore emerged as a key question (Edwards et al. 2018; Leibold et al. 2022), particularly in the context of global changes. With communities increasingly exposed to disturbances and reorganization (Tylianakis et al. 2008), and with multiple documented cases of evolution in this context (Bonnet et al. 2022), assessing the consequences of evolution for biodiversity remains a central challenge (Urban et al. 2016).

At first sight, evolution may be expected to favour diversity. On macroevolutionary scales, it has produced Earth's impressive biological richness through repeated speciation events. On shorter timescales, following disturbances, natural selection may promote adapted phenotypes, reversing population declines and preventing local extinctions—a process known as evolutionary rescue (Gomulkiewicz and Holt 1995) that may enhance diversity at a larger organizational scale, that is, “community rescue” (Low‐Décarie et al. 2015; Bell et al. 2019). By fostering adaptation, evolution could therefore buffer diversity loss. However, this perspective relies on the assumption that evolution generally increases the average growth rate of populations, turning population decline into population increase, an assumption that may not hold across ecological contexts (Dieckmann and Ferrière 2004; Metz et al. 2008; Rohr and Loeuille 2023). In fact, evolution can reduce abundances and even drive extinctions, both for the evolving population (evolutionary deterioration (Matsuda and Abrams 1994), evolutionary suicide (Parvinen 2005)) and at the community scale, where adapted species may exert stronger ecological pressures on others (Loeuille 2019) or when evolution directly weakens ecological interactions (Weinbach et al. 2022). The overall role of evolution in maintaining diversity thus remains ambiguous and requires closer examination.

Recent works have tackled this question in competitive systems. Particularly, the modern coexistence theory (Chesson 2000) has been instrumental to these developments, both on the theoretical (Pastore et al. 2021; Yamamichi et al. 2022) and on the empirical side (Germain et al. 2020; Hart et al. 2019). This theory proposes that coexistence between species can be enhanced when niche differences are increased or when fitness differences among species are reduced. Analysing the effects of evolution on stabilizing differences (niche differences) and competitive differences (fitness differences), Pastore et al. (2021) showed that evolved reduction in stabilizing differences played a critical role in coexistence, but depended on the breadth of environmental conditions. Yamamichi et al. (2022) showed that when fast evolution is assumed, competitive and stabilizing differences could be defined from an eco‐evolutionary point of view and be used to predict coexistence. From an empirical point of view, stabilizing and competitive differences can be assessed for coevolved and non coevolved populations to discuss the interplay of evolution and coexistence. Germain et al. (2020) applied this method to show that a native grass species *Vulpia microstachys* in California evolved higher competitive ability (reduced competitive differences) after the invasion of *Bromus hordeaceus* . Based on field experiments using aquatic plant species, Hart et al. (2019) similarly found an evolutionary reduction of competitive differences through convergent evolution, though evolution here did not change the coexistence outcome.

While such works based on competition offer interesting insights on the role of evolution for coexistence, the consequences of evolution for coexistence depend critically on the type of interaction. In mutualistic interactions, evolution may either strengthen the interaction—stabilizing coexistence—or weaken it, undermining persistence (Kiers et al. 2010; Weinbach et al. 2022; Yacine and Loeuille 2024). In trophic interactions, adaptive foraging by predators can generate negative frequency dependence, favouring rare prey and thus maintaining diversity (Kondoh 2003; Loeuille 2010a). Trophic structure is also sensitive to evolutionary change: for example, the evolution of plant defences can limit energy transfer along food chains, constraining the number of trophic levels (Loeuille and Loreau 2004; Koffel et al. 2018; Leibold et al. 2022). Next to interaction type, trade‐off types are equally important. Traits that incur allocative costs (e.g., diverting resources from maintenance or reproduction, Herms and Mattson (1992)) may reduce population densities and energy availability for higher trophic levels. Traits can also generate ecological costs (Strauss et al. 2002; Müller‐Schärer et al. 2004), where improving one interaction (e.g., stronger mutualism) simultaneously worsens another (e.g., greater susceptibility to herbivory). For instance, some plant defences are efficient against generalist herbivores while positive or attractive towards specialist herbivores (Müller‐Schärer et al. 2004), or plant traits that attract pollinators can also attract herbivores in various contexts (Gómez 2003; Ramos and Schiestl 2019) Evolution of such traits creates immediate indirect ecological effects, with substantial consequences for coexistence.

Recent advances in coexistence theory, especially in the structural approach, offer new tools to study species maintenance given environmental perturbations, within a tractable but generalizable framework (Rohr et al. 2014; Saavedra et al. 2017; Medeiros et al. 2021; Lepori et al. 2024). This approach partitions coexistence across scales. Thereafter, we will use two measures of coexistence. “Overall coexistence” at community scale and “resistance” at the species level, based on how close the considered species is to extinction. Because evolution directly alters growth and interaction rates, it shifts coexistence boundaries in predictable ways. In simple systems, these concepts can be illustrated graphically, making the framework accessible and intuitive. These illustrations are based on a geometric space defined by species intrinsic growth rates, in which vectors embodying interactions are displayed (Rohr et al. 2014; Saavedra et al. 2017). Because growth rates and interaction vectors are also directly linked to benefits and costs of phenotypic traits, the graphical approach can also be used to discuss the likely direction of evolution, thereby linking eco‐evolutionary dynamics and coexistence in a unified geometrical space.

The aim of this article is to provide such graphical tools for exploring the interplay between evolution and coexistence. As for most graphical approaches, considered systems are simplified (typically two species). While graphical tools on evolution and coexistence also exist, for example based on isoclines (Vasseur et al. 2011) or more commonly using stabilizing and competitive differences (Pastore et al. 2021; Yamamichi et al. 2022), most approaches focus on competitive interactions (though see Spaak and De Laender (2020) for possible extensions). Basing our analysis on structural stability: (i) allows us to flexibly account for any interaction type; (ii) directly displays intrinsic growth rates and ecological interactions. This last point is key for our approach as it facilitates the discussion of trade‐offs and the links with empirical systems. As expected from previous works, effects of evolution on coexistence are variable. Nevertheless, the graphical approach (i) clarifies the conditions under which evolution is likely to have positive or negative effects and (ii) highlights the empirical information most critical for evaluating these outcomes. After outlining the method, we illustrate its applicability with two case studies: (i) the evolution of plant defences and its consequences on plant–herbivore communities (based on Müller‐Schärer et al. (2004)) and (ii) the well‐documented role of body size in shaping intrinsic growth rates and interaction strengths in competitive and predatory contexts.

## 2 Geometric Approach

We build our geometric approach in two steps. First, following earlier articles on structural stability (Rohr et al. 2014; Saavedra et al. 2017), we show potential coexistence based on the intrinsic growth rates of the two species, given their interactions (Figure 1A). Second, we highlight that vector length and position help to predict the likely direction of evolution (e.g., selection or counter‐selection of traits linked to $\alpha_{11}$ on Figure 1B,C respectively), based on adaptive dynamics techniques. Once this likely direction is identified, implications of evolution for coexistence can be analysed (arrows on Figure 1B,C).

**FIGURE 1:**
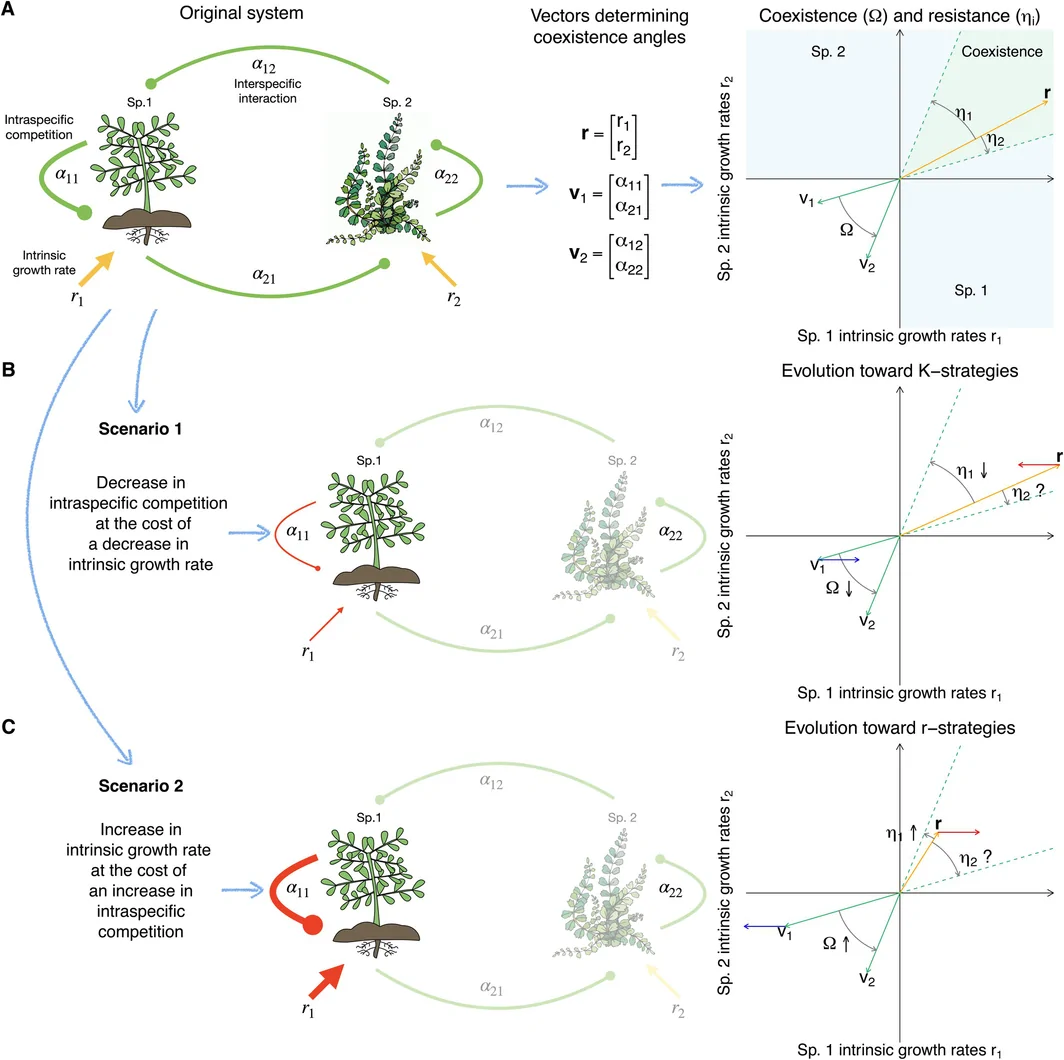
Combining the geometric approach to coexistence and to evolution. Panel A illustrates the geometric approach to coexistence for a system of two competing species. The dynamics of the system are given by species intrinsic growth rates $r_{i}$ (yellow lines), the intraspecific and interspecific interactions $\alpha_{ij}$ (green lines). For a competing system, one has $\alpha_{ij}<0$, indicated by a round tip and $r_{i}>0$, indicated by an arrow tip. From species interactions and intrinsic growth rates, the three vectors (r, $v_{1}$, $v_{2}$) determining coexistence are derived, which, in turn, govern the geometric representation of coexistence (feasibility cone), the community‐level overall coexistence potential Ω and the species‐level resistance $\eta_{i}$ metrics of coexistence. In the 2‐dimensional space made by species 1 and 2 intrinsic growth rates ($r_{1}$ and $r_{2}$), coexistence require that the vector of intrinsic growth rates r (in yellow) is inside the green cone generated by vectors $v_{1}$ and $v_{1}$ defined by ecological interactions. Therefore, angles $\eta_{1}$ and $\eta_{2}$ between r and the border of green cone quantify species resistance, that is, the perturbation needed to lose the species, while the angle Ω quantifies the overall potential of coexistence. (B, C) We consider two possible scenarios of evolution. In panel B, species 1 is likely to evolve towards K‐strategies, that is, a reduction in competition at the cost of a reduction in intrinsic growth rates. Reduction in both parameters are represented by the red lines. These reductions are then translated in the geometric representation of coexistence, by the blue and red arrow, respectively. Effects of evolution on coexistence metrics are then reported using arrows next to coexistence components Ω, $\eta_{1}$ and $\eta_{2}$. Panel C conversely shows a case where evolution favours r‐strategies, that is, an increase in intrinsic growth rate at the cost of an increase in intraspecific competition. Finally, assuming the adaptive dynamic framework of evolution, vectors and angles also provide the likely direction of evolution (derivation in the text), the K‐strategy for the angle and vectors depicted in panel B and the r‐strategy for the ones in panel C.

### 2.1 Coexistence

More precisely, let us start by presenting how panel A of Figure 1 is constructed. Consider a (general) Lotka‐Volterra model of two interacting species (Volterra 1931; Case 2000):

(1) $$\left\{\begin{array}{c} \frac{dN_{1}}{dt}=N_{1}\cdot \left(r_{1}+\alpha_{11}\cdot N_{1}+\alpha_{12}\cdot N_{2}\right)\\ \frac{dN_{2}}{dt}=N_{2}\cdot \left(r_{2}+\alpha_{21}\cdot N_{1}+\alpha_{22}\cdot N_{2}\right) \end{array}\right.$$

The intrinsic growth rate of species i is noted $r_{i}$, while $\alpha_{\mathrm{ii}}$ corresponds to its intraspecific competition, assumed to be negative. Parameters $\alpha_{\mathrm{ij}}$ correspond to the ecological effects of species j on species i, and their signs determine the types of interactions (trophic, competitive, or mutualistic).

Figure 1A shows how the conditions leading to the coexistence of two species in competition can be represented geometrically (MacArthur 1972; Vandermeer 1975; Pielou 1977; Logofet 1993; Saavedra et al. 2017). Briefly, the interaction coefficients $\alpha_{\mathrm{ij}}$ determine the two vectors $v_{1}={\left[\alpha_{11},\alpha_{21}\right]}^{t}$ (quantifying ecological effects of species 1 on itself and on the other species) and $v_{2}={\left[\begin{array}{c} \alpha_{12},\alpha_{22} \end{array}\right]}^{t}$ (ecological effects of species 2). In turn, these vectors determine the so‐called domain of feasibility (green cone on Figure 1A), that is, the set of intrinsic growth rates $r={\left[\begin{array}{c} r_{1},r_{2} \end{array}\right]}^{t}$, leading to the existence of a positive equilibrium. Whenever vector r is inside the green cone, there exists an equilibrium at which both species have strictly positive abundances. To get coexistence, one needs stability on top of the feasibility, which is ensured whenever $\alpha_{11}\cdot \alpha_{22}-\alpha_{12}\cdot \alpha_{21}>0$ (MacArthur and Levins 1967; Case 2000). This inequality can also be geometrically represented by the directed angle between the vectors $v_{1}$ and $v_{2}$, and this angle called Ω, must then fulfil $0^{\circ }<\Omega <180^{\circ }$. Being directly linked to the size of the feasibility domain, the angle Ω is here a measure of the overall coexistence potential, our community scale metric. This metric can be complemented by a species‐scale measure η that captures the resistance of each species to disturbances. The directed angle between r and the two borders of the feasibility domain, denoted by $\eta_{1}$ and $\eta_{2}$, indeed quantifies the resistance of corresponding species towards extinction, as smaller angles indicate that the corresponding species is close to extinction and that any perturbation of parameters would lead to its loss. These angles are set with a positive sign when r is inside the domain of feasibility, and the resistance measure goes to zero when the corresponding species goes towards extinction. In the example of Figure 1A, Species 2 is much closer to extinction than Species 1.

The overall coexistence potential and resistance angles for two species can be computed as follows:

(2) $$\begin{array}{c} \cos\Omega =\frac{v_{1}\cdot v_{2}}{∥v_{1}∥∥v_{2}∥},\;\sin\eta_{1}=-\frac{r\times v_{2}}{∥r∥∥v_{2}∥},\;\text{and}\;\\ \sin\eta_{2}=-\frac{v_{1}\times r}{∥r∥∥v_{1}∥}. \end{array}$$

Note that while Figure 1 is based on competition, this approach can be used for any type of ecological interaction (supporting Information S1, Figures S2, S3, S4 and S5 show other interaction types).

### 2.2 Evolution

Now that we have represented conditions of coexistence, we aim at depicting the effects of eco‐evolutionary dynamics using graphical tools in the same space ($r_{1}-r_{2}$). The superposition of ecological and evolutionary components would then allow us to display how eco‐evolutionary dynamics affect coexistence, as in Figure 1B,C. We model eco‐evolutionary dynamics using adaptive dynamics (Dieckmann and Law 1996; Geritz et al. 1998). We stress that the application of the graphical tool does not rely on adaptive dynamics. Other frameworks (e.g., quantitative genetics) could be used to determine the direction of evolution, or simply, when empirical information is available on trait variations, it can be directly used within the framework (as detailed below). Adaptive dynamics relies on various simplifications (separation of timescales (slow evolution), purely phenotypic approach), but it has several advantages that are here useful for our argument: (1) fitness is properly defined, based on the underlying ecology (Metz et al. 1992); (2) this definition includes frequency and density dependence selection (Dieckmann and Ferrière 2004). This is especially critical when looking at the ecological implications of evolution, as we do here.

In adaptive dynamics, fitness is defined as the per capita growth rate of a rare mutant of phenotype $x_{m}$ in an environment determined by the resident population x (Metz et al. 1992). Here, we do not aim at following the whole dynamics of a given phenotype, but rather to understand the direction of natural selection at a given point in time, and how it likely affects coexistence for future generations. We assume that the evolving species is species 1 and that mutations alter a phenotype directly linked to a given interaction (e.g., $\alpha_{11}$ or $\alpha_{12}$) (see also Loeuille (2010b)). Such mutations can be linked to different kinds of trade‐offs. We assume that mutations either incur allocative costs, $r_{1}$ being directly affected, or ecological costs (sensu Strauss et al. (2002)) where a benefit on an interaction will lead to costs on another. For instance, in Figure 1B,C, we consider a mutation along the r−K continuum (MacArthur and Wilson 1967; Pianka 1970), where the intraspecific competition of the species $\alpha_{11}$ is traded against intrinsic fecundity $r_{1}$, which corresponds to an allocative trade‐off. Trade‐offs may also be temporally absent (e.g., a trait has a cost in terms of specialist enemies that are not present, or an allocative cost in a context where much energy is available). In such instances, our method still applies, and evolution is simply directional.

Figure 1B shows such an example in which traits that decrease intraspecific competition are likely selected. As $\alpha_{11}$ becomes less negative, this affects the position of the vector $v_{1}$ (blue arrow on vector $v_{1}$) with an allocative cost on $r_{1}$ (red arrow on vector r). To see why the geometry of this panel corresponds to a likely selection of competitive ability, let us compute the associated fitness gradient. Remembering that fitness is defined by the *per capita* growth rate of a rare mutant of trait $\alpha_{11}^{m}$ in a resident population $\alpha_{11}$ (Metz et al. 1992), the fitness gradient, which gives the direction of evolution (Dieckmann and Law 1996), can be written:

(3) $${\left.\frac{\partial \omega \left(\alpha_{11}^{m},\alpha_{11}\right)}{\partial \alpha_{11}^{m}}\right|}_{\alpha_{11}^{m}=\alpha_{11}}=\frac{dr_{1}}{d\alpha_{11}}+\frac{\sin\left(\eta_{1}\right)}{\sin\left(\Omega \right)}\cdot \frac{∥r∥}{∥v_{1}∥}.$$

K‐strategies are favoured, higher (less negative) $\alpha_{11}$, when this gradient is positive, that is, if the trade‐off strength $\frac{dr_{1}}{d\alpha_{11}}>-\frac{\sin\left(\eta_{1}\right)}{\sin\left(\Omega \right)}\cdot \frac{∥r∥}{∥v_{1}∥}$ and counter‐selected otherwise. Intriguingly, this means that adaptive dynamics can also be partly captured by the graphical approach. The ratio of angles and vector length $\frac{\sin\left(\eta_{1}\right)}{\sin\left(\Omega \right)}\cdot \frac{∥r∥}{∥v_{1}∥}$ (right‐hand side), that is, geometry of coexistence, indeed determines a threshold in the allocative cost (left‐hand side) for selection or counter‐selection. In Figure 1B, the angle $\eta_{1}$ being quite large and the vector r having a large norm compared to the vector $v_{1}$, selection of K‐strategies is likely, as the contrary would require extremely high costs. Conversely, Figure 1C shows an example where selection of r‐strategies is expected, $\eta_{1}$ being small and the vector $v_{1}$ having a large norm compared to the vector r. Full derivation is provided in Supporting Information S2.

In the case of increases in $\alpha_{11}$ (Figure 1B), evolution harms coexistence both when measured based on overall coexistence (Ω) and when measured as resistance of species 1 ($\eta_{1}$). Not surprisingly, the opposite applies for counterselected $\alpha_{11}$ (Figure 1C). Effects of evolution on species 2 resistance ($\eta_{2}$) is undetermined, as it depends on the relative magnitude of the blue and red arrows and on the amplitude of vectors r and $v_{1}$. Nevertheless, we can further compute its derivative relative to the intraspecific competition, again based on the geometry of coexistence and the trade‐off strength,

(4) $$\frac{d\eta_{2}}{d\alpha_{11}}=\frac{\alpha_{21}}{∥v_{1}{∥}^{2}}-\frac{r_{2}}{∥r{∥}^{2}}\cdot \frac{dr_{1}}{d\alpha_{11}}$$

This expression gives a second threshold in trade‐off strength, $\frac{\alpha_{21}\cdot ∥r{∥}^{2}}{r_{2}\cdot ∥v_{1}{∥}^{2}}$ determining the change in species 2 resistance. Full derivation is provided in Supporting Information S2. Note that effects of evolution on $\eta_{2}$ is undetermined because we are here in a competition context. For a positive effect of species 1 on species 2, $\alpha_{21}>0$ and a positive intrinsic growth rate for species 2, $r_{2}>0$, given the previous equation, changes of $\eta_{2}$ throughout evolution is systematically determined (see Figure S3 panels B and D).

Our graphical approach, here shown for competitive interactions and allocative trade‐offs, can be extended to any types of pairwise interactions (competition, antagonistic and mutualistic) and trade‐off types (allocative and ecological trade‐offs), or even no trade‐off. In Supporting Information S2, we show the full case when the phenotype affects $\alpha_{11}$ and $r_{1}$, in S3 the phenotype affects $\alpha_{12}$ and $r_{1}$, in S4 the phenotype affects $\alpha_{11}$ and $\alpha_{12}$ and finally in S5 the phenotype affects $r_{1}$, $\alpha_{12}$ and $\alpha_{21}$.

## 3 Applying the Graphical Approach to Empirically Informed Cases

While the geometrical approach we propose can be used to illustrate and understand theoretical cases (as in the previous part), importantly, it is also possible to apply it to situations in which empirical information is known. More precisely, the data needed to apply the approach are: (1) current variations in the phenotypic trait; (2) likely trade‐offs associated with the trait (e.g., effects on intrinsic growth rates or ecological interactions). We stress that while quantified information on these two aspects allows better prediction, it may not be necessary. Qualitative, or semi‐quantitative information (e.g., are some variations larger or smaller compared to others) already allow some insights, as in the two examples we take below (Figures 2 and 3). These examples consider two types of traits that are particularly well‐documented: plant defences and body size.

**FIGURE 2:**
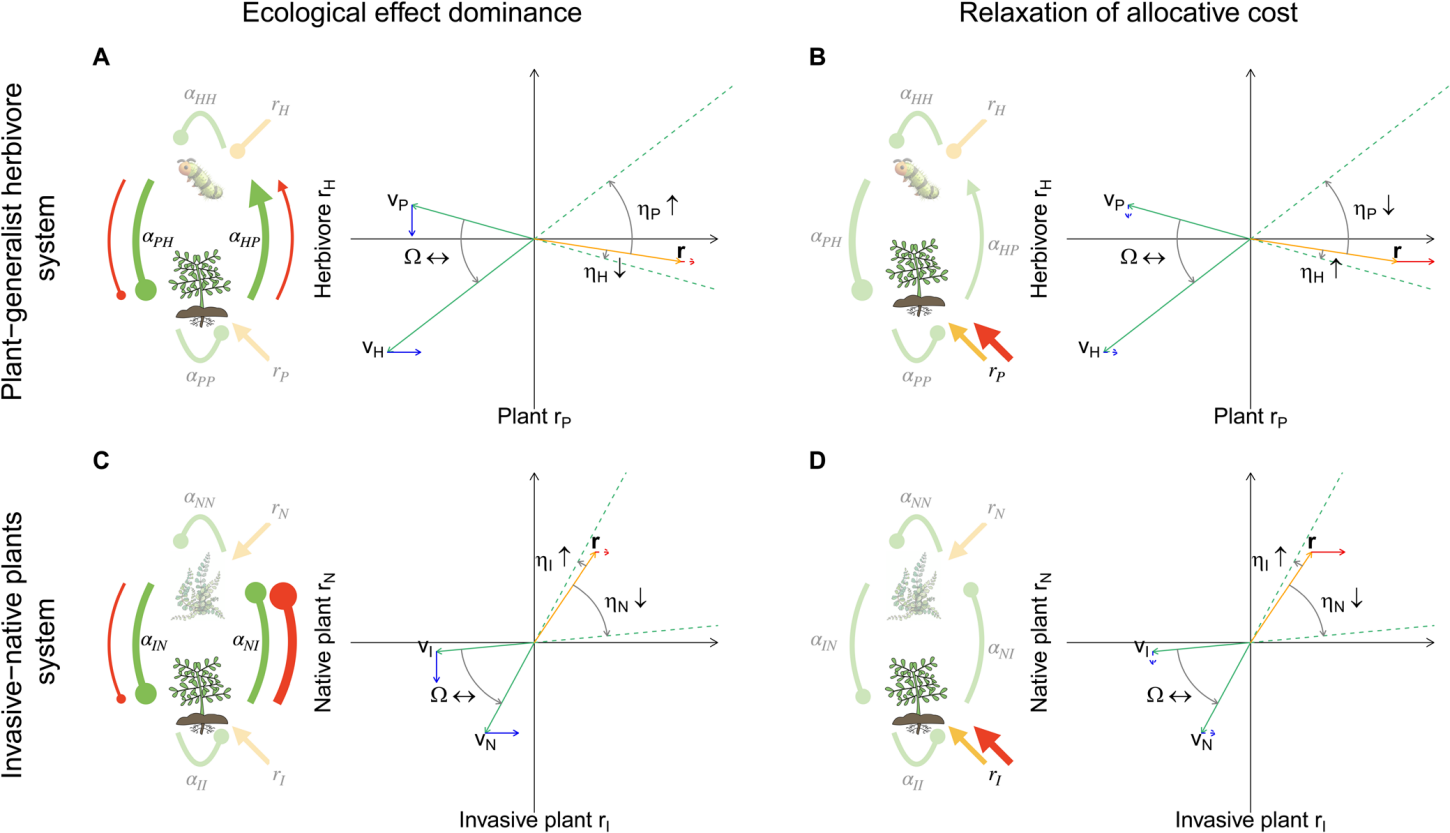
Effects of evolution of plant defences, based on empirical arguments proposed by (Müller‐Schärer et al. 2004). Here, evolution is considered on two different types of defence traits: Qualitative and quantitative, within the new range of the invasive species. Panels A and B show effects on coexistence from the herbivore–invasive plant perspective, while panels C and D show the effects of evolution from a native–invasive perspective. Strength of ecological vs. allocation costs are contrasted, the former being dominant in A and C, the latter in B and D. In panel A, evolution decreases the trophic interaction between the invasive plant and generalist herbivore, depicted by the red lines and the two blue arrows. In panels B and D, evolution increases invasive plant intrinsic growth rate, depicted by the red lines and the red arrow. In panel C, evolution decreases the competitive advantage of the native plant on the invasive one, while increasing the one from the invasive plant on the native one, depicted by the red lines and the two blue arrows. Note that effects on coexistence depend on the relative costs in the plant–herbivore perspective, not in the invasive–native perspective.

**FIGURE 3:**
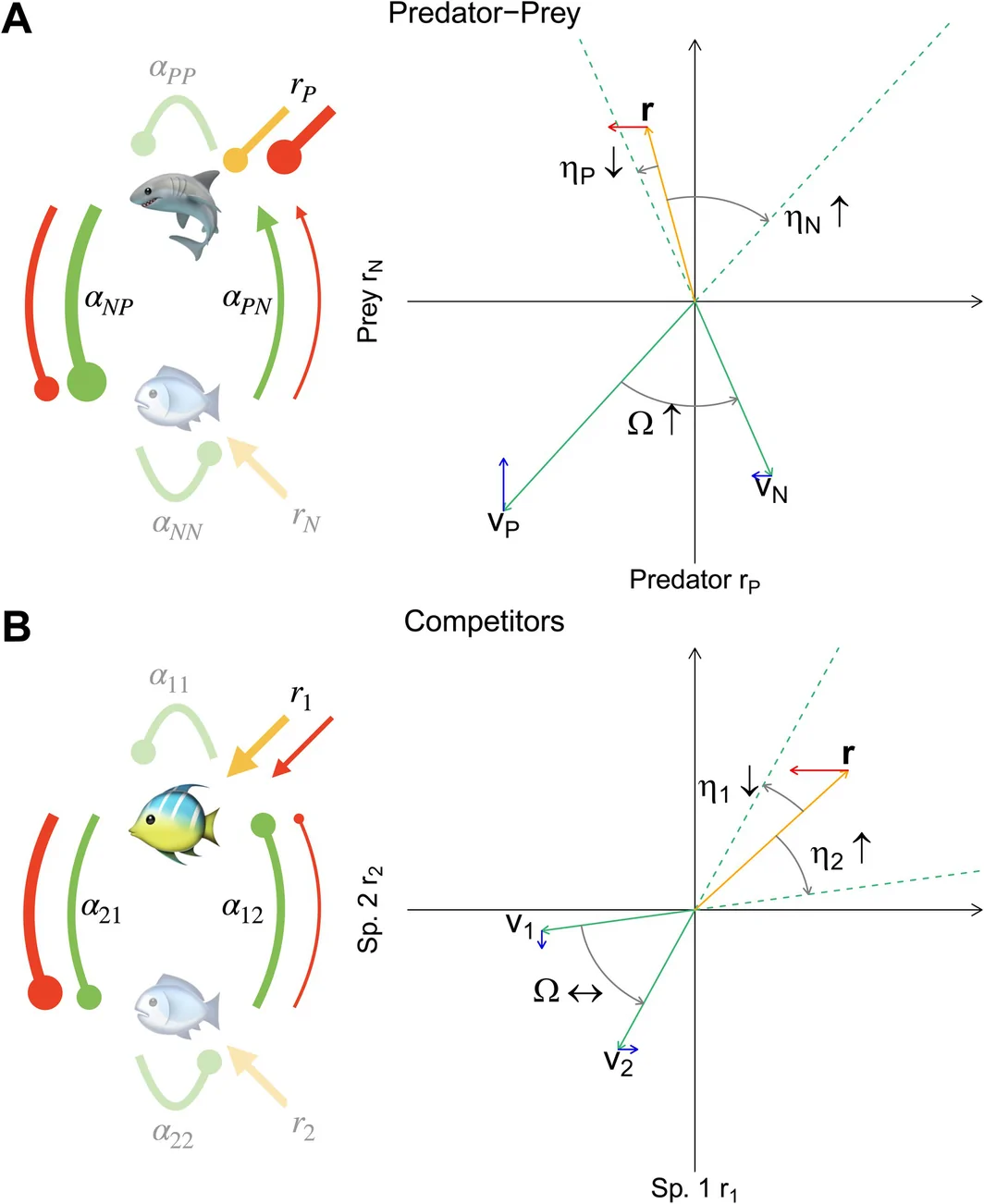
Effects of the evolution of body size (as measured by adult body mass) on species coexistence. Panel A trophic. Based on the evolution of lowered body size in harvested fishes. For example, reduction in cod (predator) body size based on (Olsen et al. 2004). We here assume that such an evolution decreases the cod attack rate (red line and blue arrows on interaction vectors), while also modifying Malthusian intrinsic growth rates (based on metabolic theory, red line and arrow). Evolution here enhances overall coexistence, while making the predator more vulnerable. Panel B competition. We assume intense interference competition, selecting for higher body sizes. Such an evolution modifies the competitive hierarchy (red line and blue arrows on interaction vectors) while reductions of the Malthusian intrinsic growth rate are again based on the metabolic theory of ecology (red line and arrow). Note that we here assume that interaction impacts are low compared to growth variations, so that evolution increases the evolving species vulnerability, while leaving overall coexistence potential unchanged.

### 3.1 Evolution of Plant Defences and the Control of Invasive Species

Our first illustrative example is based on the ideas expressed in Müller‐Schärer et al. (2004) that review empirical information on the evolution of defences of invasive plant species in their new range. We here summarize the main points. The article proposes to distinguish two types of defence traits. Based on empirical evidence, *qualitative defences* (toxins) are assumed to be efficient against generalist enemies, but at the cost of attracting specialists. Specialists indeed adapt and are not affected by these defences due to their tight co‐evolution with the plant species and may even use these toxins as attraction cues or, after ingestion, as their own defences. The selection of qualitative defences therefore follows ecological trade‐offs, balancing interactions with specialist vs. generalist herbivores. Because specialists are often absent in the new range, qualitative defences become cost‐free and higher levels can be selected there (Müller‐Schärer et al. 2004). *Quantitative defences* (e.g., tannins, lignin) are supposed to involve direct costs in terms of intrinsic growth rate (Herms and Mattson 1992) and/or competitive ability (Müller‐Schärer et al. 2004), therefore involving both allocative costs and ecological costs. If, as stated above, qualitative defences have been selected, herbivore pressure should be low on the invasive species, and quantitative defences should be counter‐selected (Müller‐Schärer et al. 2004). This counter‐selection would result in higher intrinsic growth rates and higher competitive ability for the evolving invasive species.

While this empirical case may seem a bit complex (two different types of defences, with multiple impacts on ecological interactions and intrinsic growth rates), our graphical approach can accommodate this type of complexity. To show this, we now plug this empirically‐based analysis into our graphical approach. While a 3D plot of all three species would be doable, for clarity, we separate the effects of evolution into two parts: (i) on the invasive plant—generalist herbivore system (Figure 2A,B) and (ii) on the invasive plant—local competitors (Figure 2C,D).

The graphical analysis highlights the crucial role of certain aspects of the problem in analysing the consequences of evolution for the maintenance of the system. More specifically, these consequences depend on the relative strength of ecological and allocative costs of defences (left vs. right column of Figure 2).

First, consider the effects of the evolution of defences on the (invasive) plant‐herbivore system. The evolution of qualitative defences decreases the impact of generalist herbivores on plants, thereby leading to the movements of vectors $v_{P}$ and $v_{H}$ (blue arrows on Figure 2A,B). Because quantitative defences are counter‐selected, the relaxation of associated allocation costs leads to higher plant intrinsic growth rates (red arrow on Figure 2A,B). Based on the ecological effects alone (blue arrows), we expect that evolution does not change much the overall coexistence at the community scale (as measured by Ω). However, consequences for herbivore and plant compartments vastly differ depending on the relative size of ecological and allocation costs. When ecological effects are dominant (Figure 2A), evolution mostly harms the herbivore population (lower resistance $\eta_{H}$) while enhancing plant robustness (higher $\eta_{P}$). In such situations, direct effects of the evolution of qualitative defences dominate; the trophic interaction is smaller, which leads to a reduction in energy transfer detrimental to the herbivore population. Conversely, if the dominant effect is the relaxation of allocative costs (i.e., higher $r_{P}$, as in Figure 2B), evolution fosters herbivore resistance (increase in $\eta_{H}$) and decreases plant resistance (lower $\eta_{P}$). Indeed, in such a situation, the counter‐selection of quantitative defences allows an increase in plant basic productivity that increases the herbivore compartment through bottom‐up effects.

When looking at the consequences of the evolution of invasive‐native competition, ecological vs. allocative costs now influence the outcome similarly (Figure 2C,D). Remembering that the counter‐selection of quantitative defences not only relaxes allocation costs (red arrow on Figure 2C,D), but also increases the competitive ability of the invasive species (blue arrows on Figure 2C,D), consequences for the system persistence can be directly analysed using the graphical approach. When ecological costs are dominant (Figure 2C), evolution leads to a decrease in native resistance (lower $\eta_{N}$) and to an increase in invasive resistance (higher $\eta_{I}$), as expected, based on the increased competitive asymmetry. This effect is reinforced by the relaxation of allocative costs in Figure 2D. The invasive plant cumulates two advantages: higher competitive ability and higher intrinsic growth rates, further reducing native resistance and threatening the maintenance of the system.

Our graphical approach is used here to provide possible scenarios for future coexistence. However, it also serves to point out critical aspects that should be investigated to get a more in‐depth understanding. Here, for instance, the outcome on the trophic side (plant‐herbivore) depends on the relative size of ecological and allocative costs, while the competition outcome is qualitatively the same. While the assessment of trade‐offs is notoriously difficult, note that quite a lot of information exists on both types of costs (e.g., Herms and Mattson (1992), Müller‐Schärer et al. (2004)), so that informed scenarios can be proposed when this information is available. If not, our approach highlights that getting information on these costs could be a priority. Furthermore, while Figure 2 proposes that Ω may be left unchanged throughout evolution, this guess is based on that we have no information on the asymmetry of trait impacts on the interactions (i.e., we assumed similar sizes for blue arrows). Getting further information on such impacts would refine possible consequences of evolution.

### 3.2 Ecological Effects of Body Size Evolution

Body size (most often measured as adult body mass) is likely the best documented phenotype in ecology. Variations in body size are known to affect many aspects of life history, metabolic constraints and ecological interactions (Peters 1986). Based on these empirical observations, it is possible to use the graphical approach to better understand how the evolution of body size influences coexistence. To do so, we build on two examples: (i) the often reported reduction in body size due to intense fishing (e.g., in cod populations (Olsen et al. 2004)), (ii) the general idea that larger body size offers a competitive advantage in interference competition (Persson 1985). These two examples are chosen because they allow us to illustrate different types of interactions (trophic and competitive respectively) as well as a phenotype that incurs various benefits and costs (hence complex trade‐off shapes).

Olsen et al. (2004) suggests a fast evolution of cod populations in Newfoundland due to intense fishing. Given the high rate of mortality due to direct exploitation, individuals that mature early have a selective advantage. This leads to a fast reduction of the age at maturity, accompanied by a reduction in adult body size. Such an evolution is not an exception and has been observed in various harvested or hunted populations (Barot et al. 2004; Grift et al. 2003; Conover et al. 2005). Our graphical approach allows the investigation of the possible consequences of the evolved reduction in size for predator–prey coexistence. Following the metabolic theory of ecology (Brown et al. 2004), we assume that mortality rates are increased (i.e., faster decline) when body size becomes smaller (hence negative effects on $r_{p}$). We also assume that trophic interactions are based on relative sizes of predators and prey (Brose et al. 2006; Naisbit et al. 2012; Li et al. 2023). Assuming that the cod population is initially adapted to the size of its prey, evolved reductions in body size decrease attack rates.

These assumptions lead to the graphical outcome reported on Figure 3A. Since we assumed that the trophic interaction is here relaxed due to changes in predator body size, the impact on interaction vectors is arguably asymmetric. Indeed, only a fraction of consumed prey is incorporated in predator variations (Lindeman 1942), making predator vs. prey impacts intrinsically asymmetric (Neutel and Thorne 2014). Under this hypothesis, overall coexistence is enhanced by evolution (as measured by angle Ω). Effects on the resistance of the two trophic levels depend on whether changes in body size affect more strongly the predator intrinsic growth rate (red arrow) or the trophic interaction (blue arrows). In the first case, predator resistance ($\eta_{P}$) is increased while prey resistance $\eta_{N}$ decreases. Higher predator intrinsic growth rate indeed increases top‐down pressure on prey population, while predators benefit from lower mortality. In the second case, as displayed on panel A, predator resistance is decreased while prey resistance is increased. Due to fishing pressures, the size of the predator is then increasingly maladapted to the consumption of the prey species and top‐down control is reduced.

Effects of variations in body size in a competitive context can be similarly worked out. Imagine that competition is asymmetric, large body sizes being favoured (Persson 1985). For instance, in Trinidadian guppy populations, a long‐term study on four different populations highlighted how larger body size is systematically linked to increased competition on smaller phenotypes (Griffiths et al. 2020). Assuming intense competition, such larger body sizes are selected for Species 1, it is possible to infer the consequences of such variations using our framework. More precisely, still based on allometric relationship based on the metabolic theory of ecology (Brown et al. 2004), larger body sizes are here associated with lower intrinsic growth rates. As shown on Figure 3B, evolution shifts vectors $v_{1}$ and $v_{2}$, so that overall coexistence (angle Ω) is not changed in a systematic way. Concerning species resistance, the outcome again depends on the relative effects of body size variations on the demographic (red arrow) vs. competitive interactions (blue arrows). If demographic components vary more with body size, as displayed on 3B, evolution harms the existence of the evolving species while if movement of vector $v_{2}$ (relaxed competitive effects of Species 2 on Species 1) is important, resistance of species 1 is enhanced.

## 4 Discussion

Understanding the intricate relationships between evolution and the diversity and structure of ecological communities is at the heart of eco‐evolutionary studies (Edwards et al. 2018; Hart et al. 2019). Given the many observations of evolution recently reported under current changes (Bonnet et al. 2022), this understanding is urgently needed to better forecast the future of biodiversity and its implications for ecosystem functioning. While the method we propose here is based on just a few species, we believe that it may help by providing a graphical approach that explicitly links (i) evolutionary dynamics, here analysed through either adaptive dynamics approaches or detailed empirical information; (ii) coexistence theory, based on structural stability. While mathematical analysis incorporating these two frameworks is certainly possible (e.g., Lepori et al. (2024)), our graphical approach offers the possibility to be readily usable by non‐theoreticians, thereby possibly facilitating the discussion between experimental/empirical observations and theoretical developments of eco‐evolutionary dynamics.

Concerning the evolutionary part, we show that the fitness gradient can be decomposed into two parts: one that captures the costs of the phenotypic trait at hand (for instance, how the intrinsic growth rate changes when the trait varies (allocation costs)) and the other that can be easily expressed in terms of basic intrinsic growth rates and interaction intensity. This second part can be directly drawn within our graphical approach, that is grounded in the representation of interaction vectors within a space defined by species intrinsic growth rates. Even though assessing the amplitude of evolutionary costs is notoriously difficult (Mauricio 1998; Reznick et al. 2000), the graphical approach may still give the likely direction of evolution. For instance, Figure 1 (panels B and C) shows two situations in which selection and counter‐selection of the trait are likely, in the sense that only extreme costs could prevent these directions. We emphasize that in between these two extreme situations, the determination of the likely direction of evolution may be more ambiguous. We also stress that while we here developed our evolutionary approach based on adaptive dynamics approaches, it is conceivable to use it under other frameworks. For instance, in many quantitative genetic studies, fitness is expressed based on variations of ecological characteristics of the species and of its interactions (Taper and Case 1992; Norberg et al. 2012). In such conditions, the fitness gradient may similarly be computed, and the approach we propose applied.

Once the direction of evolution is found out, it can be displayed directly within the graphical approach, as arrows affecting the three vectors that determine coexistence. The recent development of structural stability analysis (Rohr et al. 2014; Saavedra et al. 2017; Lepori et al. 2024) can then be used to determine how the general coexistence (Ω angle) and the vulnerabilities of the two species ($\eta_{i}$ angles) are changed throughout evolution. In some cases, the role of evolution is not ambiguous. For instance, the example based on evolution of defences in invasive plants show that vulnerabilities change in a systematic way, both for the invasive and for the resident species (Figure 2). The same case, however, suggests that general coexistence may not be affected (angle Ω is mostly unchanged). We acknowledge however that this statement is based on the idea that evolution affects the two interaction vectors roughly the same way. To provide a more precise assessment, one would need to uncover the relative amplitude of evolutionary effects on the various interaction rates, which may be empirically difficult. Still, the graphical approach proposes a reference scenario, that can then be tested experimentally or through an analysis of empirical datasets.

While theoretical developments based on our framework require a formalization of evolution (e.g., using adaptive dynamics or quantitative genetics), applications can be based on relevant empirical observations, as shown by examples on Figures 2 and 3. This requires information on phenotype variations for the evolving species, and on the costs or benefits of these trait variations on species intrinsic growth rate or interaction intensity (i.e., information on trade‐offs). More precise information on these elements improves possible predictions, but the examples we take here suggest that even qualitative or semi‐quantitative information (e.g., ideas of the dominant effects) may suffice to investigate some aspects of coexistence. We stress here that while we framed the article based on evolutionary processes, variations of phenotypes due to plasticity or epigenetics could similarly be considered, as long as associated benefits and costs are known. While data requirements may be limiting, our examples show that they exist for some traits. Other cases could have been used here. Fragata et al. (2025), for instance, experimentally study the competition of two spider mite species on tomato plants that had accumulated cadmium or not. They reconstruct the structural stability space in each case and highlight the implications of evolution in each case, clearly showing that all information is present to apply the framework. Another relevant example concerns the evolution of resident plant species *Vulpia microstachys* following the invasion of Californian grasslands by *Bromus hordeaceus* (Germain et al. 2020; Sakarchi and Germain 2023). While these works rely on modern coexistence theory, they contain information on species intrinsic growth rates and competitive interactions that could be directly implemented in the graphical approach we propose here. Interestingly evolution here affects competitive interactions while no phenotypic variation is clear (Germain et al. 2020). This suggests that information on phenotypic variations may not be fully needed to apply the framework. Beyond these competitive cases, our framework allows for the investigation of various other interaction types, which may be key to get a more integrative view of coexistence.

The method also needs the assessment of interaction rates and of intrinsic growth rates of species. Information may, however, be limiting, especially regarding the intensity of ecological interactions. While the knowledge of ecological interaction intensity is sparse, many methods have been developed throughout the years, from experimental approaches dating back to Paine (1992) to the reconstruction of the variability of feeding links within food webs (Paine 1980; Rooney et al. 2006; Goldwasser and Roughgarden 1993). Methods based on steady state conditions and species allometry (including ecosim/ecopath approaches) are vastly used to assess relative interactions (De Ruiter et al. 1995; Jacquet et al. 2016) (see Gauzens et al. (2023) for a recent package development). When time series of abundances are available, more advanced techniques can also be used (Sugihara 1994; Nguyen et al. 2025). If a precise assessment of ecological interactions cannot be achieved, the method may still allow discussing plausible scenarios and offer a first take on the question at hand. For instance, in the various empirical examples we propose in the text, a precise assessment of the interaction is not included, but we simply use available information on reported evolutions to propose possible effects on coexistence. More precise assessment of the various ingredients would, however, allow us to refine the proposed outcomes.

The framework we propose has several important limitations. First, as a graphical approach, it is suited to study subsystems containing few species. Typically, the coexistence space is easy to draw in two dimensions (all figures presented here) and could be extended to a third dimension to depict three species at the same time. For more complex systems (diffuse co‐evolution *sensu* Janzen (1980)), a graphical approach may not be suitable. A possible way around is to split the complex system into different subparts and to try to understand various pieces of the puzzle. For instance, in the invasive‐resident‐herbivore system we use here, based on empirical inputs from Müller‐Schärer et al. (2004), we split the system into two 2‐species subsystems, to allow a better visualization of the effects of evolution within guilds and among trophic levels. For higher diversity scenarios, however, numerical simulations are likely needed, and the graphical approach becomes limited. We also restate that the method is used to tackle effects and direction of evolution at a given point in time, not to follow the evolutionary dynamics until their outcome. While this limit is quite restrictive from a purely theoretical perspective (e.g., understanding the eventual effects of evolution, close to the evolutionary attractor), the framework may still help to work out the effects of evolution on coexistence, based on experiments or empirical data, for the next generations of the considered species. In turn, this information may be used and considered for a better understanding and management of the systems at hand. Second, our graphical approach relies on linear Lotka‐Volterra models. While including non‐linearity in the structural stability approach to coexistence has been proposed (Cenci and Saavedra 2018), non‐linear demographic models would make the use of a graphical approach much harder, as it does for other graphical approaches (e.g., isocline approaches Vasseur et al. (2011)). Third, our approach does not relate to the classic stabilizing vs. competitive difference plots (Pastore et al. 2021; Yamamichi et al. 2022) and, therefore, does not discuss the effects of evolution based on components of the modern coexistence theory. We, however, believe this is more an advantage than a limitation. Instead of relying on aggregate properties, our approach allows a direct representation of changes in intrinsic growth rates and/or interaction strengths and links them to the overall coexistence potential and to species resistance. This may facilitate links with empirical works.

A development of proper graphical tools is often instrumental to improve the link between theory and its applications. Lotka‐Volterra systems of competition and predation, while often mathematically tractable, are often taught based on isocline graphs, and these isocline graphs allow an intuition that serves to make theory accessible to a broad spectrum ranging from bachelor students to non‐theoretician researchers in ecology. Even among theoreticians, simplified graphical approaches help to clarify key mechanisms, as exemplified by the use of ZNGI (zero net growth isoclines) approaches throughout decades (Tilman 1982; Koffel et al. 2021). On the evolution theory side, adaptive dynamics is a good example of a modelling technique that succeeded not only based on its theoretical merits (e.g., an ecologically based definition of fitness Metz et al. (1992) and of the related variations of traits Dieckmann and Law (1996)), but also because a graphical approach was proposed to visualize and understand evolutionary dynamics based on PIPs (pairwise invasibility plots, Geritz et al. (1998)). We therefore believe that a graphical approach that link evolution and coexistence may help to bridge a gap between theoretical developments and empirical assessments of the question, and hope that the framework we propose here will be useful to fellow researchers interested in these questions.

## Author Contributions

Nicolas Loeuille and Rudolf P. Rohr contributed equally to the manuscript.

## Funding

This work was supported by Schweizerischer Nationalfonds zur Förderung der Wissenschaftlichen Forschung (CRSII5 202290, 320030L‐227556).

## Supporting information


**Supporting Information S1:** Geometry of two species coexistence.
**Supporting Information S2:** Evolution of a phenotype affecting α11 and r1.
**Supporting Information S3:** Evolution of a phenotype affecting α12 and r1.
**Supporting Information S4:** Evolution of a phenotype affecting α11 and α12.
**Supporting Information S5:** Evolution of a phenotype affecting r1, α12, and α21.
**Figure S1:** Geometric approach to coexistence. The per capita interaction strength determine the vectors **v**_1_ and **v**_2_, while, **r** gives the intrinsic growth rates. The green cone is the domain of feasibility, that is, feasibility is granted if and only if **r** is inside this domain. The directed angles Ω determine the overall coexistence potential and stability condition, while the angles *η*_1_ and *η*_2_ are the resistance angles of Species 1 and 2, respectively.
**Figure S2:** The position of the vectors **v**_1_ and **v**_2_ determines the types of interactions between Species 1 and Species 2.
**Figure S3:** Effect of evolution of Species 1 on the structural metrics in the case of an allocative trade‐off between r1 and α11. Here, we assume that evolution relaxes intraspecific competition at the cost of a decrease in intrinsic growth rate (evolution towards a K strategy). Other cases can be obtained by simply setting accordingly the red and blue arrows, and then, deducing the direction of the black arrows.
**Figure S4:** Effect of evolution of Species 1 on the structural metrics in the case of an allocative trade‐off between r1 and α12. Here, we assume that evolution relaxes the negative effect of species 2 on species 1 (evolution towards better defences), or increase the positive effect, at the cost of a decrease in intrinsic growth rate. Other cases can be obtained by simply setting accordingly the red and blue arrows, and then, deducing the direction of the black arrows.
**Figure S5:** Effect of evolution of Species 1 on the structural metrics in the case of an allocative trade‐off between α11 and α12. Here, we assume that evolution relaxes the negative effect of species 2 on species 1 (evolution towards better defences), or increase the positive effect, at the cost of an increase in the intraspecific competition. Other cases can be obtained by simply setting accordingly the red and blue arrows, and then, deducing the direction of the black arrows.

## Data Availability

Data sharing not applicable to this article as no datasets were generated or analysed during the current study.
