# Supplementary material for: Graphical Approach of the Interplay of Eco‐Evolutionary Dynamics and Coexistence
Source: Ecol Lett. 2026 Nov 13;29(11):e70470. doi: 10.1111/ele.70470 (PMC13768472; doi:10.1111/ele.70470)
Supplement: Supplementary file 1 [file ELE-29-0-s001.pdf]

Supporting Information for

A graphical approach of the interplay of eco-evolutionary dynamics and coexistence Nicolas

Loeuille and Rudolf P. Rohr
nicolas.loeuille@sorbonne-universite.fr and rudolf.rohr@unifr.ch

section.1 section.2 subsection.2.1 subsection.2.2 section.3 subsection.3.1 subsection.3.2 section.4

S1 Geometry of two species coexistence 1

S2 Evolution of a phenotype affecting α_{11} and r_1 6

S3 Evolution of a phenotype affecting α_{12} and r_1 10

S4 Evolution of a phenotype affecting α_{11} and α_{12} 14

S5 Evolution of a phenotype affecting r_1, α_{12}, and α_{21} 18

S1 Geometry of two species coexistence

We consider the Lotka-Volterra model for two interacting species of abundance N_1 and N_2 (Volterra, 1931; Case, 2000). It is given by the following set of differential equations:

$$\begin{cases} \dfrac{\mathrm{d}N_1}{\mathrm{d}t} = N_1 \cdot (r_1 + \alpha_{11} \cdot N_1 + \alpha_{12} \cdot N_2) \\ \dfrac{\mathrm{d}N_2}{\mathrm{d}t} = N_2 \cdot (r_2 + \alpha_{21} \cdot N_2 + \alpha_{22} \cdot N_2) \end{cases} \tag{S1}$$

The parameters are the intrinsic growth rates r_i of species i, and the *per capita* interaction of species j on species i denoted by α_{ij}. These parameters can be encapsulated into the vector $\mathbf{r}$ of intrinsic growth rates and the matrix $\mathbf{A}$ of *per capita* interactions:

$$\mathbf{r} = \begin{bmatrix} r_1 \\ r_2 \end{bmatrix} \quad \text{and} \quad \mathbf{A} = \begin{bmatrix} \alpha_{11} & \alpha_{12} \\ \alpha_{21} & \alpha_{22} \end{bmatrix} = \begin{bmatrix} | & | \\ \mathbf{v}_1 & \mathbf{v}_2 \\ | & | \end{bmatrix}. \tag{S2}$$

The interaction matrix can be decomposed into two column vectors $\mathbf{v}_1$ and $\mathbf{v}_2$ capturing the *per capita* effect of species 1 and 2 on their interactor and on themselves.

To allow coexistence in such a system, two conditions need to be fulfilled: (i) feasibility and (ii) stability (Volterra, 1931; MacArthur and Levins, 1967; Vandermeer, 1975; Logofet, 1993; Case, 2000). Feasibility corresponds to the existence of a non-trivial, i.e., positive, equilibrium point. Such a point denoted by $(N_1^* > 0, N_2^* > 0)$ must, therefore, be a solution of the following linear equation.

$$\mathbf{r} + \mathbf{A} \cdot \begin{bmatrix} N_1^* \\ N_2^* \end{bmatrix} = \begin{bmatrix} 0 \\ 0 \end{bmatrix} \tag{S3}$$

The stability conditions, in turn, grant that the non-trivial equilibrium, if it exists, is locally stable. The condition for stability is given by

$$\alpha_{11} \cdot \alpha_{22} - \alpha_{12} \cdot \alpha_{21} > 0. \tag{S4}$$

Note, that in a two species Lotka-Volterra model, local stability is equivalent to global stability.

The aim of the structural approach to coexistence is to provide a geometric interpretation to the feasibility (equ. S3) and stability (equ. S4) equations. Regarding the feasibility constraints, instead of solving directly the equation (S3), we represent the set of intrinsic growth rates ($\mathbf{r}$) leading to a positive solution. This is achieved by rewriting equation (S3) as

$$\mathbf{r} = -\mathbf{A} \cdot \begin{bmatrix} N_1^* \\ N_2^* \end{bmatrix} = -\mathbf{v}_1 \cdot N_1^* - \mathbf{v}_2 \cdot N_2^*. \tag{S5}$$

That is, as feasibility requires $N_1^* > 0$ and $N_2^* > 0$, the set of $\mathbf{r}$ leading to a feasible solution are the positive linear combinations of the vectors $-\mathbf{v}_1$ and $-\mathbf{v}_2$. This set of feasible $\mathbf{r}$, called the feasibility domain, can be represented geometrically as a cone (figure S1) in the two-dimensional $r_1 - r_2$ space, where the x-axis is the intrinsic growth rate of species 1 and the y-axis is the intrinsic growth rate of species 2. The borders of the feasibility domain are determined by the *per capita* interaction strengths. The smallest angle between the two vectors $\mathbf{v}_1$ and $\mathbf{v}_2$, denoted by Ω, quantifies the size of the feasibility domain and therefore the potential of a feasibility.

In turn, the stability condition also has a geometric representation. The term $\alpha_{11} \cdot \alpha_{22} - \alpha_{12} \cdot \alpha_{21}$ in the stability condition, is equivalent to the cross product between the two vectors $\mathbf{v}_1$ and $\mathbf{v}_2$, and therefore, proportional to the sinus of Ω:

$$\alpha_{11} \cdot \alpha_{22} - \alpha_{12} \cdot \alpha_{21} = \mathbf{v}_1 \times \mathbf{v}_2 = \sin \Omega \cdot \|\mathbf{v}_1\| \cdot \|\mathbf{v}_2\|. \tag{S6}$$

That is, stability is equivalent to a positive cross product between the vectors $\mathbf{v}_1$ and $\mathbf{v}_2$,

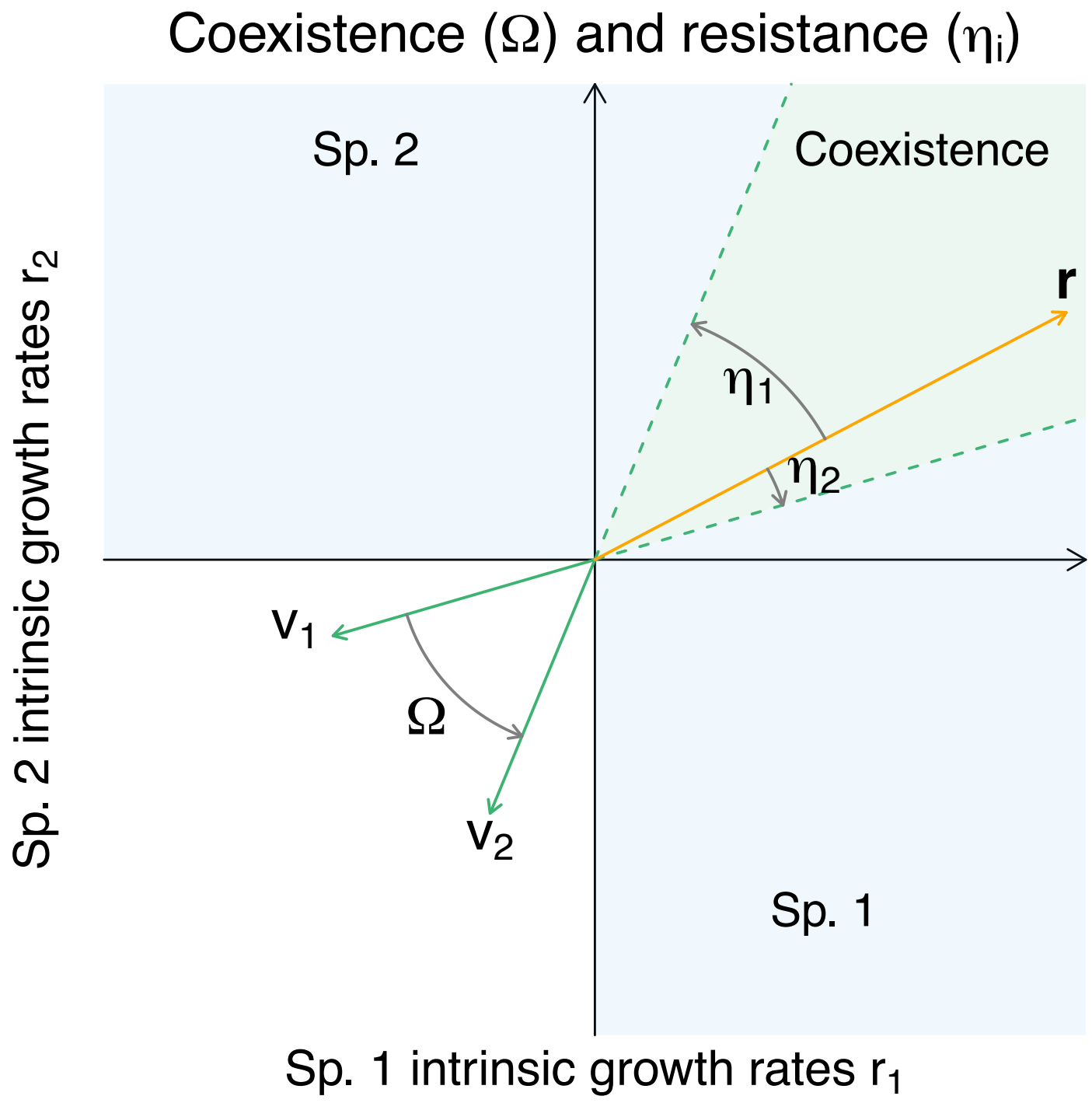

Figure S1: Geometric approach to coexistence. The per capita interaction strength determine the vectors $\mathbf{v_1}$ and $\mathbf{v}_2$, while, $\mathbf{r}$ gives the intrinsic growth rates. The green cone is the domain of feasibility, i.e., feasibility is granted if and only if $\mathbf{r}$ is inside this domain. The directed angles Ω determine the overall coexistence potential and stability condition, while the angles η_1 and η_2 are the resistance angles of species 1 and 2, respectively.

while a negative cross product implies instability. Therefore, to geometrically represent stability, we can add a direction to the angle Ω, i.e., turn it into a directed angle between $\mathbf{v}_1$ and $\mathbf{v}_2$. The direction of Ω is given in figure S1 by the arrow on the arc of a circle depicting Ω. The directed angle Ω is defined as the smallest angle between $\mathbf{v}_1$ and $\mathbf{v}_2$, and its sign is positive if the vector $\mathbf{v}_1$ is before the vector $\mathbf{v}_2$ when considering a counter-clockwise direction. Otherwise, the sign is negative. Consequently, stability is granted if the directed angle is positive but smaller than 180°, i.e. $0° < \Omega < 180°$.

Assuming that stability conditions are fulfilled, if the vector of intrinsic growth rates $\mathbf{r}$ falls outside the feasibility domain (green cone), at least one of the two species goes extinct. In particular, if $\mathbf{r}$ is located in one of the two blue areas (figure S1), then only species 1 or species 2 survive, while the other goes extinct. Therefore, as represented in figure S1, the two directed angles η_1 and η_2 between $\mathbf{r}$ and the border of the feasibility domain are called species resistance. Note that the sign of the directed angle η_1 is defined counter-clockwise (like Ω), while the sign of the angle η_2 is defined clockwise (opposite direction). This allows the interpretation that (1) the smaller the angle (but still positive), the closer the corresponding species is to extinction, and (2) feasibility is granted if and

only if both resistance angles are positive, while if at least one angle is negative, the corresponding species is extinct.

The overall coexistence potential angle Ω and the two resistance angles η_1 and η_2 define the metrics of coexistence and are computed as follows:

$$\cos\Omega = \frac{\mathbf{v}_1 \cdot \mathbf{v}_2}{\|\mathbf{v}_1\|\|\mathbf{v}_2\|}, \quad \sin\eta_1 = -\frac{\mathbf{r} \times \mathbf{v}_2}{\|\mathbf{r}\|\|\mathbf{v}_2\|}, \quad \text{and} \quad \sin\eta_2 = -\frac{\mathbf{v}_1 \times \mathbf{r}}{\|\mathbf{r}\|\|\mathbf{v}_1\|}. \tag{S7}$$

Under feasibility and stability conditions, the equilibrium densities can be computed from the overall coexistence potential and resistance angles, i.e., have a geometric perspective:

$$\begin{cases} N_1^* = \dfrac{-r_1 \cdot \alpha_{22} + r_2 \cdot \alpha_{12}}{\alpha_{11} \cdot \alpha_{22} - \alpha_{12} \cdot \alpha_{21}} = \dfrac{\sin(\eta_1)}{\sin(\Omega)} \cdot \dfrac{\|\mathbf{r}\|}{\|\mathbf{v}_1\|} \\ N_2^* = \dfrac{r_1 \cdot \alpha_{21} - r_2 \cdot \alpha_{11}}{\alpha_{11} \cdot \alpha_{22} - \alpha_{12} \cdot \alpha_{21}} = \dfrac{\sin(\eta_2)}{\sin(\Omega)} \cdot \dfrac{\|\mathbf{r}\|}{\|\mathbf{v}_2\|} \end{cases} \tag{S8}$$

The middle terms of the above set of equations are the classical solution of equation (S3). Now, at the denominator, we can identify the term determining the stability condition, which is equivalent to the cross product between $\mathbf{v}_1$ and $\mathbf{v}_2$, i.e, proportional to the sinus of Ω. While, the terms at the two numerators are the same as the cross product between $\mathbf{v}_2$ and $\mathbf{r}$ and between $\mathbf{v}_1$ and $\mathbf{r}$, respectively. That is, the two numerators are proportional to the resistance angles. All that together leads to the right terms of equation set (S8).

Finally, note that this geometric approach can be applied to any type of interaction, as demonstrated in figure S2. The $r_1 - r_2$ space can be decomposed into four orthants. We can always assume negative density dependence of a species on itself; that is, the intraspecific interaction can be assumed to be negative $\alpha_{11} < 0$ and $\alpha_{22} < 0$. This implies that the vector $\mathbf{v}_1$ can only be in orthant II and III, while $\mathbf{v}_2$ can only be in orthant III and IV. This leads to four combinations, as illustrated by panels S1.A to S1.D describing all types of pairwise interactions.

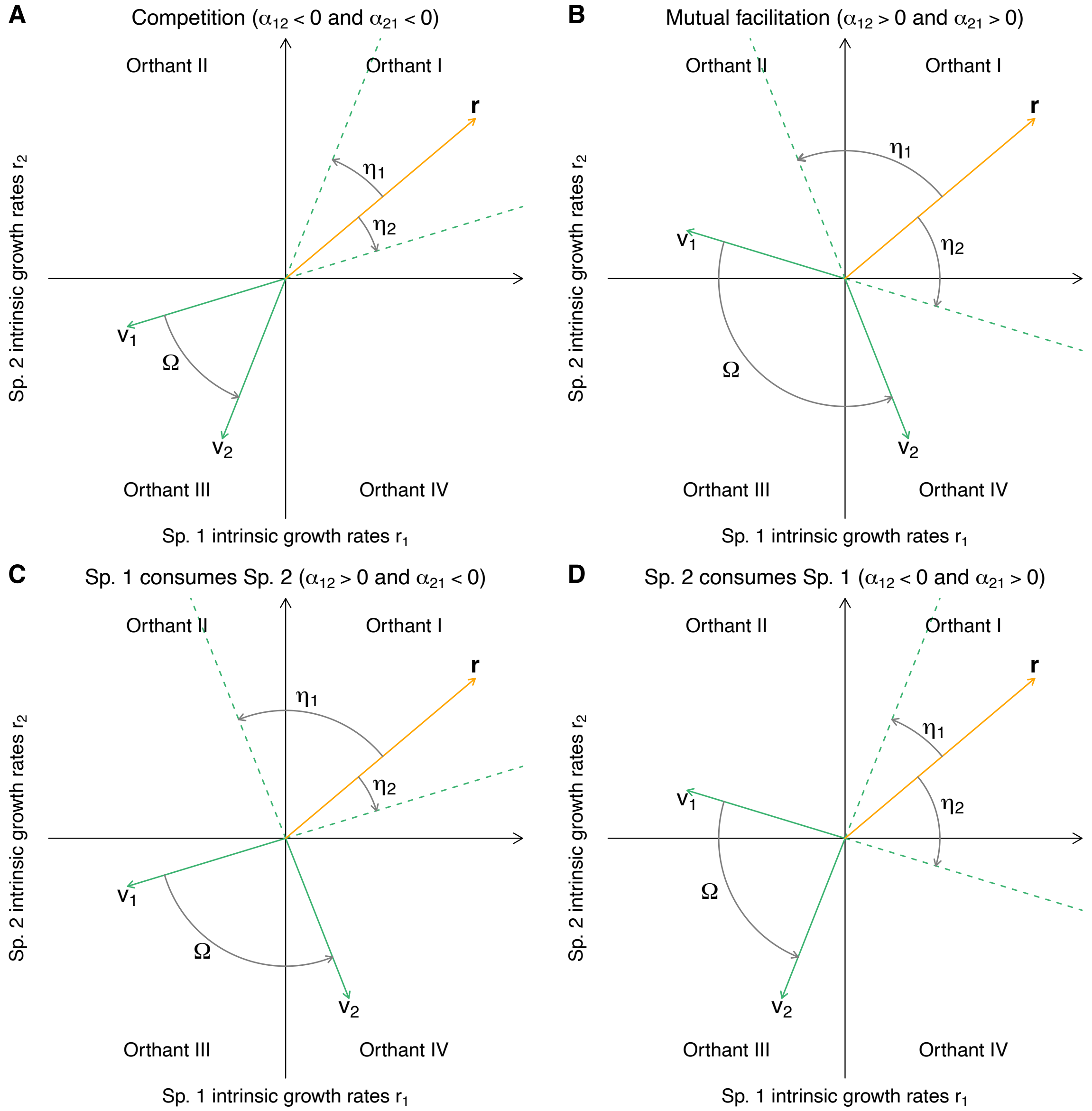

Figure S2: The position of the vectors $\mathbf{v}_1$ and $\mathbf{v}_2$ determines the types of interactions between species 1 and species 2.

S2 Evolution of a phenotype affecting α_{11} and r_1

Here, we assume that the trait z of species 1, which is under selection, impacts both the intrinsic growth rate $r_1(z)$ and intraspecific competition $\alpha_{11}(z)$. Figure S3 shows the graphical analysis of the consequences of evolution on the geometry of coexistence for the four possible combinations of interactions. In this figure, we assume that evolution relaxes intraspecific competition at the cost of intrinsic growth rate, which relates, for example, to the classical $r - K$ theory (MacArthur and Wilson, 1967). That is, evolution toward K-strategies results in a simultaneous decrease in r_1 (red arrow) and increase in α_{11} (that therefore becomes less negative, blue arrow on vector $\mathbf{v}_1$). Other cases can be obtained simply be first choosing the appropriate direction of the red and blue arrows, and then deducing the direction of the black arrows. The figure shows, for this scenario, that the effects of evolution on overall coexistence potential Ω and on species 1 resistance η_1 are systematically determined by the type of interaction that species 1 has with species 2. However, the effect on species 2 resistance η_2 is more difficult to determine graphically, as both the vector $\mathbf{r}$ and $\mathbf{v}_1$ are displaced through evolution and, in some cases, in the same direction (panels A and C). In such instances, as mathematically demonstrated below, the impact of evolution on η_2 is determined by the combination of the trade-off strength, $\mathrm{d}r_1(z)/\mathrm{d}z$ / $\mathrm{d}\alpha_{11}(z)/\mathrm{d}z$, and the vectors $\mathbf{r}$ and $\mathbf{v}_2$.

These graphical results can be mathematically proven by computing the derivative, relative to the trait z, of the overall coexistence potential Ω and the two resistance angles η_1 and η_2. Moreover, the selection gradient can also be related to the geometry of coexistence. We start by deriving the selection gradient, and then we compute $\mathrm{d}\Omega/\mathrm{d}z$, $\mathrm{d}\eta_1/\mathrm{d}z$, and $\mathrm{d}\eta_2/\mathrm{d}z$.

The invasion fitness of a rare mutant of trait z_m in a resident population of trait z is given by its per capita growth rate when rare, the resident population being at equilibrium (Metz et al., 1992), which results in

$$\omega(z_m, z) = r_1(z_m) + \alpha_{11}(z_m) \cdot N_1^* + \alpha_{12} \cdot N_2^*. \tag{S9}$$

The direction of trait evolution is then given by the selection gradient, a positive selection gradient leading to the selection of higher trait values, while a negative gradient conversely leads to smaller trait values (Dieckmann and Law, 1996). Given the definition of fitness above, the selection gradient equals:

$$\left.\frac{\partial \omega(z_m, z)}{\partial z_m}\right|_{z_m \to z} = \frac{\mathrm{d}r_1}{\mathrm{d}z} + \frac{\mathrm{d}\alpha_{11}}{\mathrm{d}z} \cdot N_1^* = \frac{\mathrm{d}r_1}{\mathrm{d}z} + \frac{\mathrm{d}\alpha_{11}}{\mathrm{d}z} \cdot \frac{\sin(\eta_1)}{\sin(\Omega)} \cdot \frac{\|\mathbf{r}\|}{\|\mathbf{v}_1\|}. \tag{S10}$$

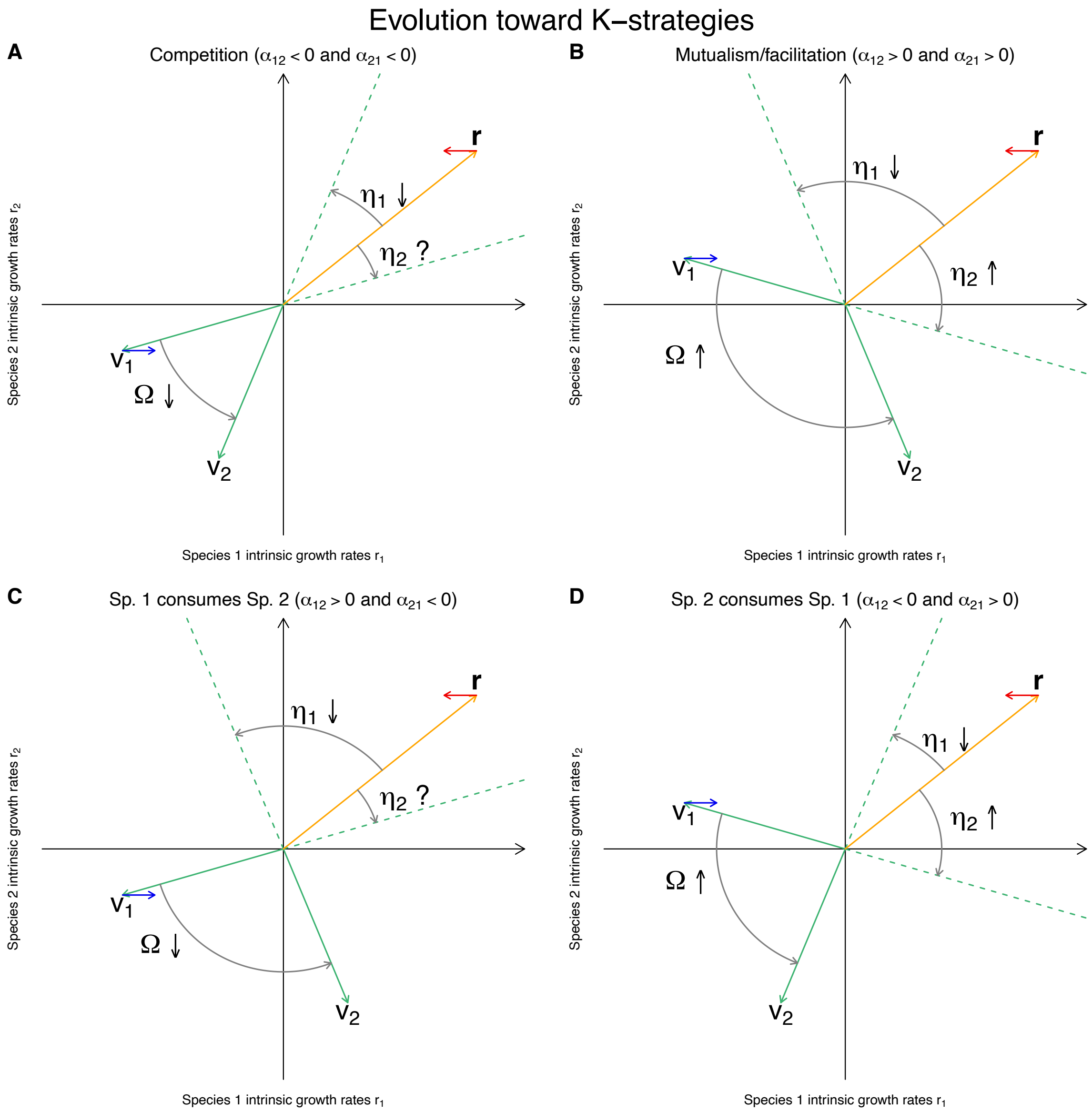

Figure S3: Effect of evolution of species 1 on the structural metrics in the case of an allocative trade-off between r_1 and α_{11}. Here, we assume that evolution relaxes intraspecific competition at the cost of a decrease in intrinsic growth rate (evolution toward a K strategy). Other cases can be obtained by simply setting accordingly the red and blue arrows, and then, deducing the direction of the black arrows.

Two cases for the direction of evolution occurs. First, one can assume an allocative trade-off between $r_1(z)$ and α_{11}, i.e., the sign of $\mathrm{d}r_1(z)/\mathrm{d}z$ / $\mathrm{d}\alpha_{11}(z)/\mathrm{d}z < 0$ is negative. In such case, the direction of evolution is determined the strength of this trade-off and the geometric of coexistence given by the angles Ω and η_1, as well as the length of the two vectors, the vectors $\mathbf{r}$ and $\mathbf{v}_1$. In the second case, where no trade-off is assumed, the signs of $\mathrm{d}r_1(z)/\mathrm{d}z$ and $\mathrm{d}\alpha_{11}(z)/\mathrm{d}z$ are the same, and therefore, the direction of evolution is determined by their sign.

The effects of evolution on the different metrics of coexistence are given by:

$$\begin{cases} \dfrac{\mathrm{d}\Omega}{\mathrm{d}z} = \dfrac{\alpha_{21}}{\|\mathbf{v}_1\|^2} \cdot \dfrac{\mathrm{d}\alpha_{11}}{\mathrm{d}z} \\ \\ \dfrac{\mathrm{d}\eta_1}{\mathrm{d}z} = \dfrac{r_2}{\|\mathbf{r}\|^2} \cdot \dfrac{\mathrm{d}r_1}{\mathrm{d}z} \\ \\ \dfrac{\mathrm{d}\eta_2}{\mathrm{d}z} = \dfrac{\alpha_{21}}{\|\mathbf{v}_1\|} \cdot \dfrac{\mathrm{d}\alpha_{11}}{\mathrm{d}z} - \dfrac{r_2}{\|\mathbf{r}\|} \cdot \dfrac{\mathrm{d}r_1}{\mathrm{d}z} \end{cases} \tag{S11}$$

Note that $\mathrm{d}\Omega/\mathrm{d}z = \mathrm{d}\eta_1/\mathrm{d}z + \mathrm{d}\eta_2/\mathrm{d}z$, which is coherent as, $\Omega = \eta_1 + \eta_2$.

To easily relate these three equations to figure S3, we can rewrite them to make apparent the changes in $\omega(z_m, z)$, Ω, η_1, and η_2 relative to the change in α_{11}. This can also be considered equivalent to considering $z = \alpha_{11}$ and, consequently, r_1 being a function of α_{11}. This leads to

$$\begin{cases} \left.\dfrac{\partial \omega(\alpha_{11}^m, \alpha_{11})}{\partial \alpha_{11}^m}\right|_{\alpha_{11}^m \to \alpha_{11}} = \dfrac{\mathrm{d}r_1}{\mathrm{d}\alpha_{11}} + \dfrac{\sin(\eta_1)}{\sin(\Omega)} \cdot \dfrac{\|\mathbf{r}\|}{\|\mathbf{v}_1\|} \\ \\ \dfrac{\mathrm{d}\Omega}{\mathrm{d}\alpha_{11}} = \dfrac{\alpha_{21}}{\|\mathbf{v}_1\|^2} \\ \\ \dfrac{\mathrm{d}\eta_1}{\mathrm{d}\alpha_{11}} = \dfrac{r_2}{\|\mathbf{r}\|^2} \cdot \dfrac{\mathrm{d}r_1}{\mathrm{d}\alpha_{11}} \\ \\ \dfrac{\mathrm{d}\eta_2}{\mathrm{d}\alpha_{11}} = \dfrac{\alpha_{21}}{\|\mathbf{v}_1\|^2} - \dfrac{r_2}{\|\mathbf{r}\|^2} \cdot \dfrac{\mathrm{d}r_1}{\mathrm{d}\alpha_{11}} \end{cases} \tag{S12}$$

The first equation shows that α_{11} is selected if and only if $\sin(\eta_1)/\sin(\Omega) \cdot \|\mathbf{r}\|/\|\mathbf{v}_1\| > -\,\mathrm{d}r_1/\mathrm{d}\alpha_{11}$. That is, the geometry of coexistence (the second term of this equation) determines a threshold for selection and counter-selection in the trade-off case (i.e. when $\mathrm{d}r_1/\mathrm{d}\alpha_{11} > 0$). The second equation demonstrates that the evolution of α_{11} and Ω are in the same direction when the effect of species 1 on species 2 is positive ($\alpha_{21} > 0$), as illustrated by panels B and D in figure S3, while it goes in the opposite direction when species 1 negatively affects species 2 ($\alpha_{21} < 0$), as illustrated by panels A and C. The third equation demonstrates that whether the evolution of α_{11} and η_1 are in the same or opposite direction is determined by the sign of $r_2 \cdot \mathrm{d}r_1/\mathrm{d}\alpha_{11}$. As, on figure S3 we assumed $\mathrm{d}r_1/\mathrm{d}\alpha_{11} < 0$ and $r_2 > 0$ evolution is in the opposite direction. The reader can imagine other cases, by, for instance locating the vector $\mathbf{r}$ in the fourth orthant on panels B and D. Finally, the last equation shows that the determination of the direction of evolution of

η_2 is more tricky, as already shown in figure S3, as it will depend on the signs of $r_2 > 0$, $\alpha_{21} > 0$, and $\mathrm{d}r_1(z)/\mathrm{d}z$ / $\mathrm{d}\alpha_{11}(z)/\mathrm{d}z$, as well as on the amplitude of the vectors $\mathbf{r}$ and $\mathbf{v}_1$. In some particulars cases, as in panels B and D, the direction is uniquely determined.

S3 Evolution of a phenotype affecting α_{12} and r_1

Here, we assume that the trait z of species 1, which is under selection, impacts both the intrinsic growth rate $r_1(z)$ and interspecific interaction $\alpha_{12}(z)$. Figure S4 shows the graphical analysis of the consequences of evolution on the geometry of coexistence for the four possible combinations of interactions. In this figure, we assume that evolution either relaxes the negative effect of species 2 on species 1 (panels A and D), or increases the positive effect of species 2 on species 1 (panels B and D), both at the cost of intrinsic growth rate. Such a situation can, for instance, relate to when prey invest in defence, i.e., a reduction of the negative impact of the predator that translates to an increase in $\alpha_{12}(z)$, at the cost of a decrease in its intrinsic growth rate $r_1(z)$ (e.g. for plant defences Herms and Mattson (1992)). That is, evolution results in a simultaneous decrease in r_1 (red arrow) and increase in α_{12} (that therefore becomes less negative or more positive, blue arrow on vector $\mathbf{v}_2$). Other cases can be obtained simply be first choosing the appropriate direction of the red and blue arrows, and then deducing the direction of the black arrows. The figure shows, for this scenario, that the effects of evolution on overall coexistence potential Ω and on species 2 resistance η_2 are systematically determined. However, the effect on species 1 resistance η_1 is more difficult to determine graphically, as both vectors $\mathbf{r}$ and $\mathbf{v}_2$ are displaced through evolution and in the same direction. In such instances, as mathematically demonstrated below, the impact of evolution on η_1 is determined by the combination of the trade-off strength, $\mathrm{d}r_1(z)/\mathrm{d}z$ / $\mathrm{d}\alpha_{12}(z)/\mathrm{d}z$, and by the norms of vectors $\mathbf{r}$ and $\mathbf{v}_2$.

These graphical results can be mathematically proven by computing the derivative, relative to the trait z, of the overall coexistence potential Ω and the two resistance angles η_1 and η_2. Moreover, the selection gradient can also be related to the geometry of coexistence. We start by deriving the selection gradient, and then we compute before computing the $\mathrm{d}\Omega/\mathrm{d}z$, $\mathrm{d}\eta_1/\mathrm{d}z$, and $\mathrm{d}\eta_2/\mathrm{d}z$.

Following the same rational as in section S2, the invasion fitness of a rare mutant of trait z_m in a resident population of trait z is given by

$$\omega(z_m, z) = r_1(z) + \alpha_{11} \cdot N_1^* + \alpha_{12}(z_m) \cdot N_2^*. \tag{S13}$$

The direction of trait evolution is then given by the selection gradient

$$\left.\frac{\partial \omega(z_m, z)}{\partial z_m}\right|_{z_m \to z} = \frac{\mathrm{d}r_1}{\mathrm{d}z} + \frac{\mathrm{d}\alpha_{12}}{\mathrm{d}z} \cdot N_2^* = \frac{\mathrm{d}r_1}{\mathrm{d}z} + \frac{\mathrm{d}\alpha_{12}}{\mathrm{d}z} \cdot \frac{\sin(\eta_2)}{\sin(\Omega)} \cdot \frac{\|\mathbf{r}\|}{\|\mathbf{v}_2\|}. \tag{S14}$$

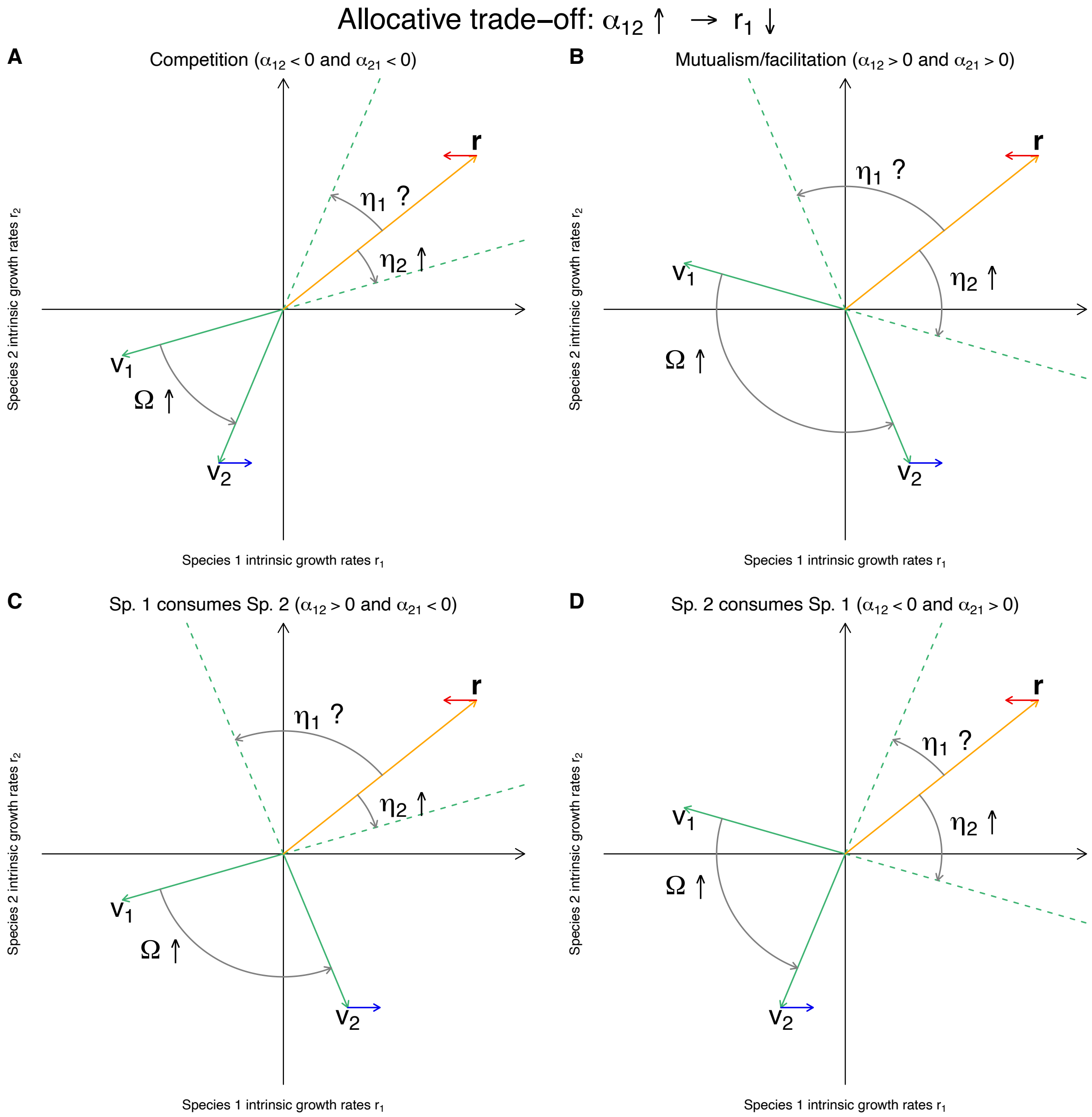

Figure S4: Effect of evolution of species 1 on the structural metrics in the case of an allocative trade-off between r_1 and α_{12}. Here, we assume that evolution relaxes the negative effect of species 2 on species 1 (evolution toward better defences), or increase the positive effect, at the cost of a decrease in intrinsic growth rate. Other cases can be obtained by simply setting accordingly the red and blue arrows, and then, deducing the direction of the black arrows.

Two cases for the direction of evolution occurs. First, one can assume an allocative trade-off between $r_1(z)$ and α_{12}, i.e., the sign of $\mathrm{d}r_1(z)/\mathrm{d}z \ / \ \mathrm{d}\alpha_{12}(z)/\mathrm{d}z < 0$ is negative. In such a case, the direction of evolution is determined by the strength of this trade-off and by the geometric of coexistence given by the angles Ω and η_2, as well as the length of vectors $\mathbf{r}$ and $\mathbf{v}_2$. In the second case, where no trade-off is assumed, the signs of $\mathrm{d}r_1(z)/\mathrm{d}z$ and $\mathrm{d}\alpha_{12}(z)/\mathrm{d}z$ are the same, and therefore, the direction of evolution is determined by their sign.

The effects of evolution on the different metrics of coexistence are given by:

$$\begin{cases} \dfrac{\mathrm{d}\Omega}{\mathrm{d}z} = -\dfrac{\alpha_{22}}{\|\mathbf{v}_2\|^2} \cdot \dfrac{\mathrm{d}\alpha_{12}}{\mathrm{d}z} \\[2ex] \dfrac{\mathrm{d}\eta_1}{\mathrm{d}z} = -\dfrac{\alpha_{22}}{\|\mathbf{v}_2\|^2} \cdot \dfrac{\mathrm{d}\alpha_{12}}{\mathrm{d}z} + \dfrac{r_2}{\|\mathbf{r}\|^2} \cdot \dfrac{\mathrm{d}r_1}{\mathrm{d}z} \\[2ex] \dfrac{\mathrm{d}\eta_2}{\mathrm{d}z} = -\dfrac{r_2}{\|\mathbf{r}\|^2} \cdot \dfrac{\mathrm{d}r_1}{\mathrm{d}z} \end{cases} \tag{S15}$$

Note that $\mathrm{d}\Omega/\mathrm{d}z = \mathrm{d}\eta_1/\mathrm{d}z + \mathrm{d}\eta_2/\mathrm{d}z$, which is coherent as, $\Omega = \eta_1 + \eta_2$.

To easily relate these three equations to figure S4, we can rewrite them to make apparent the changes in $\omega(z_m, z)$, Ω, η_1, and η_2 relative to the change in α_{12}. This can also be considered equivalent to considering $z = \alpha_{12}$ and, consequently, r_1 being a function of α_{12}. This leads to

$$\begin{cases} \left.\dfrac{\partial\omega(\alpha_{12}^m, \alpha_{12})}{\partial\alpha_{12}^m}\right|_{\alpha_{12}^m \to \alpha_{12}} = \dfrac{\mathrm{d}r_1}{\mathrm{d}\alpha_{12}} + \dfrac{\sin(\eta_2)}{\sin(\Omega)} \cdot \dfrac{\|\mathbf{r}\|}{\|\mathbf{v}_2\|} \\[2ex] \dfrac{\mathrm{d}\Omega}{\mathrm{d}\alpha_{12}} = -\dfrac{\alpha_{22}}{\|\mathbf{v}_2\|^2} \\[2ex] \dfrac{\mathrm{d}\eta_1}{\mathrm{d}\alpha_{12}} = -\dfrac{\alpha_{22}}{\|\mathbf{v}_2\|^2} + \dfrac{r_2}{\|\mathbf{r}\|^2} \cdot \dfrac{\mathrm{d}r_1}{\mathrm{d}\alpha_{12}} \\[2ex] \dfrac{\mathrm{d}\eta_2}{\mathrm{d}\alpha_{12}} = -\dfrac{r_2}{\|\mathbf{r}\|^2} \cdot \dfrac{\mathrm{d}r_1}{\mathrm{d}\alpha_{12}} \end{cases} \tag{S16}$$

The first equation shows that α_{12} is selected if and only if $\sin(\eta_2)/\sin(\Omega) \cdot \|\mathbf{r}\|/\|\mathbf{v}_2\| > -\,\mathrm{d}r_1/\mathrm{d}\alpha_{12}$. That is, the geometry of coexistence (the second term of this equation) determines a threshold for selection and counter-selection. The second equation demonstrates that the evolution of α_{12} and Ω are in the same direction as we can assume $\alpha_{22} < 0$ (intraspecific competition), which is coherent with figure S4. The fourth equation demonstrates that whether the evolution of α_{11} and η_1 are in the same or opposite direction is determined by the sign of $r_2 \cdot \mathrm{d}r_1/\mathrm{d}\alpha_{11}$. As we assume $\mathrm{d}r_1/\mathrm{d}\alpha_{11} < 0$, then if $r_2 > 0$ evolution is in the opposite direction, as illustrated in figure S4, while it is in the same direction if $r_2 < 0$. The reader can be geometrically convinced of the latter case by imagining the vector $\mathbf{r}$ to be located in the fourth orthant on panels B and D. Finally, the last equation shows that the determination of the direction of evolution of η_1 is more tricky, as already shown in figure S4, as it will depend on the signs of $r_2 > 0$, $\alpha_{21} > 0$,

and $\mathrm{d}r_1(z)/\mathrm{d}z$ / $\mathrm{d}\alpha_{11}(z)/\mathrm{d}z$, as well as on the amplitude of the vectors $\mathbf{r}$ and $\mathbf{v}_1$.

S4 Evolution of a phenotype affecting α_{11} and α_{12}

Here, we assume that the trait z of species 1, which is under selection, impacts both the intraspecific interaction α_{11} (intraspecific competition) and the interspecific interaction $\alpha_{12}(z)$ (*per capita* effect of species 2 on the per capita growth rate of species 1). Such a situation can, for instance, relate to when prey invest in defence, i.e., a reduction of the negative impact of the predator that translates to an increase in $\alpha_{12}(z)$, at the cost of an increase in its intraspecific competition (Agrawal et al., 2012) (i.e. ecological costs). Figure S5 shows the graphical analysis of the consequences of evolution on the geometry of coexistence for the four possible combinations of interactions. In this figure, we assume that evolution decreases the negative effect of species 2 on species 1 (panels A and D), or decreases the positive effect (panels B and C), but at the cost of an increase in the intraspecific competition, i.e., evolution results in a simultaneous decrease in α_{11} (that therefore becomes more negative; blue arrow on vector $\mathbf{v}_2$) and increase in α_{12} (that therefore becomes less negative or more positive; blue arrow on vector $\mathbf{v}_1$). Other cases can be obtained simply be first choosing the appropriate direction of the red and blue arrows, and then deducing the direction of the black arrows. The figure shows that the effects of evolution on both resistance angles η_1 and η_2 is uniquely determined. The evolution of η_1 is always in the same direction as of α_{12} (all panels), while the direction of η_2 evolution is opposite if species 1 as a positive effect on species 2 (panels B and D). The direction of Ω is also uniquely determined and goes in the same direction as α_{12}, when, species 1 negatively impacts species 2 (panels A and C). It is more difficult to determine graphically when the effect of species 1 on species 2 is positive, as both vectors $\mathbf{v}_1$ and $\mathbf{v}_2$ turn in the same direction. In such instances, as mathematically demonstrated below, the impact of evolution on Ω is determined by the combination of the trade-off strength, $\mathrm{d}\alpha_{12}(z)/\mathrm{d}z$ / $\mathrm{d}\alpha_{11}(z)/\mathrm{d}z$, and the vectors $\mathbf{v}_1$ and $\mathbf{v}_2$.

These graphical results can be mathematically proven by computing the derivative, relative to the trait z, of the overall coexistence potential Ω and the two resistance angles η_1 and η_2. Moreover, the selection gradient can also be related to the geometry of coexistence. We start by deriving the selection gradient, and then we compute before computing the $\mathrm{d}\Omega/\mathrm{d}z$, $\mathrm{d}\eta_1/\mathrm{d}z$, and $\mathrm{d}\eta_2/\mathrm{d}z$.

Following the same rationale as in section S2, the invasion fitness of a rare mutant of trait z_m in a resident population of trait z is given by

$$\omega(z_m, z) = r_1 + \alpha_{11}(z_m) \cdot N_1^* + \alpha_{12}(z_m) \cdot N_2^*. \tag{S17}$$

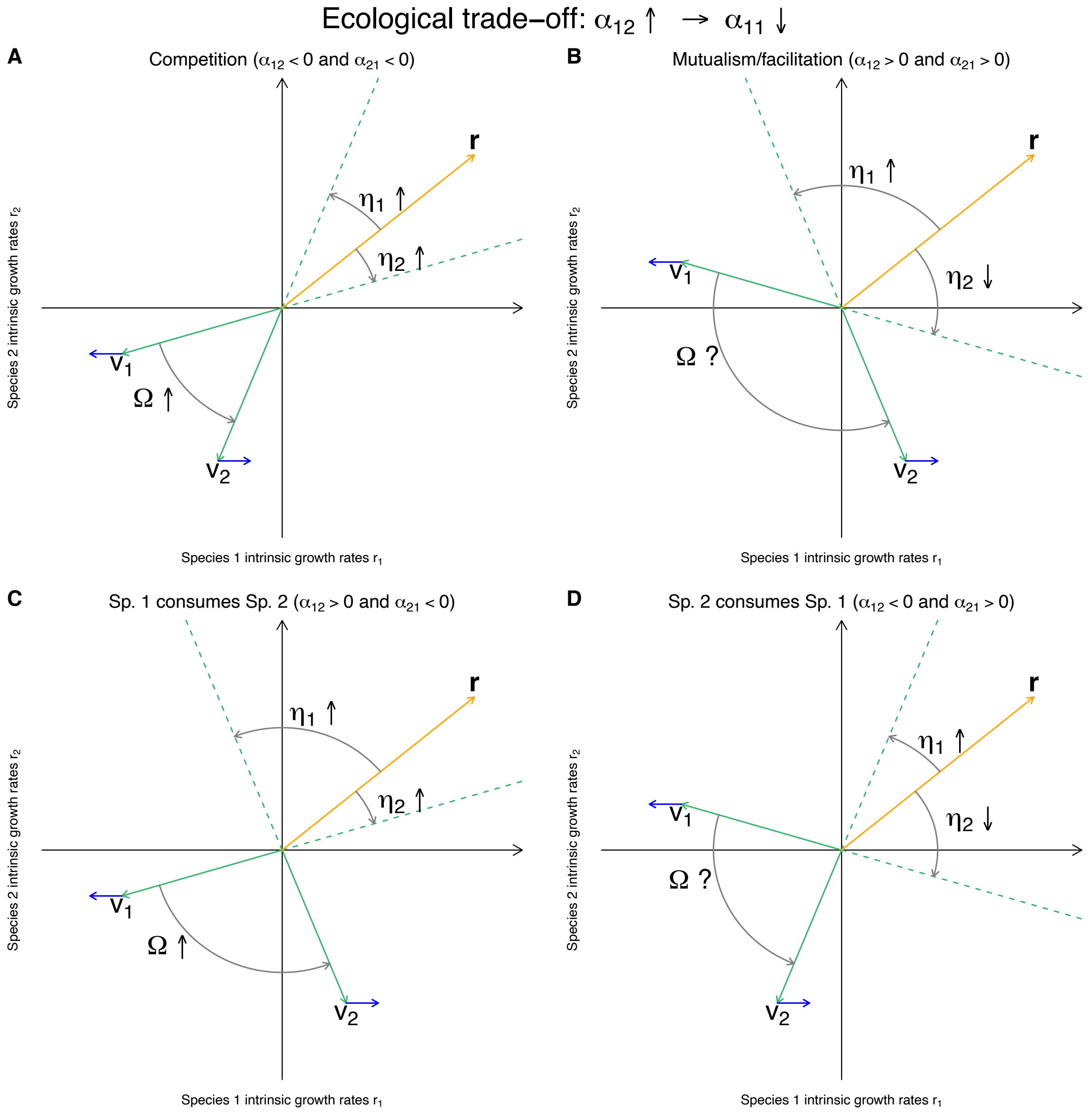

Figure S5: Effect of evolution of species 1 on the structural metrics in the case of an allocative trade-off between α_{11} and α_{12}. Here, we assume that evolution relaxes the negative effect of species 2 on species 1 (evolution toward better defences), or increase the positive effect, at the cost of an increase in the intraspecific competition. Other cases can be obtained by simply setting accordingly the red and blue arrows, and then, deducing the direction of the black arrows.

The direction of trait evolution is then given by the selection gradient

$$\left.\frac{\partial \omega(z_m, z)}{\partial z_m}\right|_{z_m \to z} = \frac{\mathrm{d}\alpha_{11}}{\mathrm{d}z} \cdot N_1^* + \frac{\mathrm{d}\alpha_{12}}{\mathrm{d}z} \cdot N_2^* = \frac{\mathrm{d}\alpha_{11}}{\mathrm{d}z} \cdot \frac{\sin(\eta_1)}{\sin(\Omega)} \cdot \frac{\|\mathbf{r}\|}{\|\mathbf{v}_1\|} + \frac{\mathrm{d}\alpha_{12}}{\mathrm{d}z} \cdot \frac{\sin(\eta_2)}{\sin(\Omega)} \cdot \frac{\|\mathbf{r}\|}{\|\mathbf{v}_2\|}. \quad \text{(S18)}$$

Two cases for the direction of evolution occur. First, one can assume an allocative trade-off between α_{11} and α_{12}, i.e., the sign of $\mathrm{d}\alpha_{12}(z)/\mathrm{d}z$ / $\mathrm{d}\alpha_{11}(z)/\mathrm{d}z < 0$ is negative. In such case, the direction of evolution is determined the strength of this trade-off and the

geometric of coexistence given by the angles Ω, η_1, and η_2, as well as the length of the vectors $\mathbf{r}$, $\mathbf{v}_1$, and $\mathbf{v}_2$. In the second case, where no trade-off is assumed, the signs of $\mathrm{d}\alpha_{11}(z)/\mathrm{d}z$ and $\mathrm{d}\alpha_{12}(z)/\mathrm{d}z$ are the same, and therefore, the direction of evolution is determined by their sign.

The effects of evolution on the different metrics of coexistence are given by

$$\begin{cases} \dfrac{\mathrm{d}\Omega}{\mathrm{d}z} = \dfrac{\alpha_{21}}{\|\mathbf{v}_1\|^2} \cdot \dfrac{\mathrm{d}\alpha_{11}}{\mathrm{d}z} - \dfrac{\alpha_{22}}{\|\mathbf{v}_2\|^2} \cdot \dfrac{\mathrm{d}\alpha_{12}}{\mathrm{d}z} \\[2ex] \dfrac{\mathrm{d}\eta_1}{\mathrm{d}z} = -\dfrac{\alpha_{22}}{\|\mathbf{v}_2\|^2} \cdot \dfrac{\mathrm{d}\alpha_{12}}{\mathrm{d}z} \\[2ex] \dfrac{\mathrm{d}\eta_2}{\mathrm{d}z} = \dfrac{\alpha_{21}}{\|\mathbf{v}_1\|^2} \cdot \dfrac{\mathrm{d}\alpha_{11}}{\mathrm{d}z} \end{cases} \tag{S19}$$

Note that $\mathrm{d}\Omega/\mathrm{d}z = \mathrm{d}\eta_1/\mathrm{d}z + \mathrm{d}\eta_2/\mathrm{d}z$, which is coherent as, $\Omega = \eta_1 + \eta_2$.

To easily relate these three equations to figure S5, we can rewrite them to make apparent the changes in $\omega(z_m, z)$, Ω, η_1, and η_2 relative to the change in α_{12}. This can also be considered equivalent to considering $z = \alpha_{12}$ and, consequently, α_{11} being a function of α_{12}. This leads to

$$\begin{cases} \left.\dfrac{\partial\omega(\alpha_{12}^m, \alpha_{12})}{\partial\alpha_{12}^m}\right|_{\alpha_{12}^m \to \alpha_{12}} = \dfrac{\mathrm{d}\alpha_{11}}{\mathrm{d}\alpha_{12}} \cdot \dfrac{\sin(\eta_1)}{\sin(\Omega)} \cdot \dfrac{\|\mathbf{r}\|}{\|\mathbf{v}_1\|} + \dfrac{\sin(\eta_2)}{\sin(\Omega)} \cdot \dfrac{\|\mathbf{r}\|}{\|\mathbf{v}_2\|} \\[2ex] \dfrac{\mathrm{d}\Omega}{\mathrm{d}\alpha_{12}} = \dfrac{\alpha_{21}}{\|\mathbf{v}_1\|^2} \cdot \dfrac{\mathrm{d}\alpha_{11}}{\mathrm{d}\alpha_{12}} - \dfrac{\alpha_{22}}{\|\mathbf{v}_2\|^2} \\[2ex] \dfrac{\mathrm{d}\eta_1}{\mathrm{d}\alpha_{12}} = -\dfrac{\alpha_{22}}{\|\mathbf{v}_2\|^2} \\[2ex] \dfrac{\mathrm{d}\eta_2}{\mathrm{d}\alpha_{12}} = \dfrac{\alpha_{21}}{\|\mathbf{v}_1\|^2} \cdot \dfrac{\mathrm{d}\alpha_{11}}{\mathrm{d}\alpha_{12}} \end{cases} \tag{S20}$$

The first equation shows that α_{12} is selected if and only if $\sin(\eta_2)/\sin(\eta_1) \cdot \|\mathbf{v}_1\|/\|\mathbf{v}_2\| > -\mathrm{d}r_{11}^{\alpha}/\mathrm{d}\alpha_{12}$. That is, the geometry of coexistence (the second term of this equation) determines a threshold for selection and counter-selection. The third equation demonstrates that the evolution of α_{12} and η_1 are in the same direction as we can assume $\alpha_{22} < 0$ (intraspecific competition), which is coherent with figure S5. The last equation demonstrates that whether the evolution of α_{12} and η_1 are in the same or opposite direction is determined by the sign of $\alpha_{21} \cdot \mathrm{d}\alpha_{12}/\mathrm{d}\alpha_{11}$. As we assume $\mathrm{d}\alpha_{12}/\mathrm{d}\alpha_{11} < 0$, then

if $\alpha_{21} > 0$ evolution is in the opposite direction, as illustrated in figure S5, while it is in the same direction if $\alpha_{21} < 0$. Finally, the second equation shows that only in the cases of $\alpha_{21} \cdot \mathrm{d}\alpha_{12}(z)/\mathrm{d}z \;/\; \mathrm{d}\alpha_{11}(z)/\mathrm{d}z < 0$, the direction of Ω evolution is uniquely determined and in the same direction as α_{12}. Otherwise, the direction of evolution is also dictated by the strength of the trade-off, $\mathrm{d}\alpha_{12}(z)/\mathrm{d}z \;/\; \mathrm{d}\alpha_{11}(z)/\mathrm{d}z$ and the sign of α_{21}.

S5 Evolution of a phenotype affecting r_1, α_{12}, and α_{21}

Empirical examples in figures 2 and 3 assume that the trait z of species 1 under selection impacts the two interspecific interactions α_{12} and α_{21}, as well as the intrinsic growth rate r_1. In the following, we derive the analytic computations for the invasion gradient and the evolution of metrics of coexistence, following the same rationale as in sections S2, S3, and S4.

The invasion fitness of a rare mutant of trait z_m in a resident population of trait z is given by

$$\omega(z_m, z) = r_1(z) + \alpha_{11} \cdot N_1^* + \alpha_{12}(z_m) \cdot N_2^*. \tag{S21}$$

We remark that, as the interaction α_{21} does not intervene in the per capita growth rate of the rare mutant, this is the exact same expression as in section S3. Then, the direction of trait evolution is then given by the selection gradient

$$\left.\frac{\partial \omega(z_m, z)}{\partial z_m}\right|_{z_m \to z} = \frac{\mathrm{d}r_1}{\mathrm{d}z} + \frac{\mathrm{d}\alpha_{12}}{\mathrm{d}z} \cdot N_2^* = \frac{\mathrm{d}r_1}{\mathrm{d}z} + \frac{\mathrm{d}\alpha_{12}}{\mathrm{d}z} \cdot \frac{\sin(\eta_2)}{\sin(\Omega)} \cdot \frac{\|\mathbf{r}\|}{\|\mathbf{v}_2\|}. \tag{S22}$$

The effects of evolution on the different metrics of coexistence are given by

$$\begin{cases} \dfrac{\mathrm{d}\Omega}{\mathrm{d}z} = -\dfrac{\alpha_{22}}{\|\mathbf{v}_2\|^2} \cdot \dfrac{\mathrm{d}\alpha_{12}}{\mathrm{d}z} - \dfrac{\alpha_{11}}{\|\mathbf{v}_1\|^2} \cdot \dfrac{\mathrm{d}\alpha_{21}}{\mathrm{d}z} \\[2ex] \dfrac{\mathrm{d}\eta_1}{\mathrm{d}z} = \dfrac{r_2}{\|\mathbf{r}\|^2} \cdot \dfrac{\mathrm{d}r_1}{\mathrm{d}z} - \dfrac{\alpha_{22}}{\|\mathbf{v}_2\|^2} \cdot \dfrac{\mathrm{d}\alpha_{12}}{\mathrm{d}z} \\[2ex] \dfrac{\mathrm{d}\eta_2}{\mathrm{d}z} = -\dfrac{r_2}{\|\mathbf{r}\|^2} \cdot \dfrac{\mathrm{d}r_1}{\mathrm{d}z} - \dfrac{\alpha_{11}}{\|\mathbf{v}_1\|^2} \cdot \dfrac{\mathrm{d}\alpha_{21}}{\mathrm{d}z} \end{cases} \tag{S23}$$

Note that $\mathrm{d}\Omega/\mathrm{d}z = \mathrm{d}\eta_1/\mathrm{d}z + \mathrm{d}\eta_2/\mathrm{d}z$, which is coherent as, $\Omega = \eta_1 + \eta_2$.

To relate these three equations to figures 2 and 3, we can rewrite them to make apparent the changes in $\omega(z_m, z)$, Ω, η_1, and η_2 relative to the change in α_{12}. This can also be considered equivalent to considering $z = \alpha_{12}$ and, consequently, α_{21} and r_1 being

a function of α_{12}. This leads to

$$\begin{cases} \left.\dfrac{\partial \omega(\alpha_{12}^m, \alpha_{12})}{\partial \alpha_{12}^m}\right|_{\alpha_{12}^m \to \alpha_{12}} = \dfrac{\mathrm{d}r_1}{\mathrm{d}\alpha_{12}} + \dfrac{\sin(\eta_2)}{\sin(\Omega)} \cdot \dfrac{\|\mathbf{r}\|}{\|\mathbf{v}_2\|} \\ \dfrac{\mathrm{d}\Omega}{\mathrm{d}\alpha_{12}} = -\dfrac{\alpha_{22}}{\|\mathbf{v}_2\|^2} - \dfrac{\alpha_{11}}{\|\mathbf{v}_1\|^2} \cdot \dfrac{\mathrm{d}\alpha_{21}}{\mathrm{d}\alpha_{12}} \\ \dfrac{\mathrm{d}\eta_1}{\mathrm{d}z} = \dfrac{r_2}{\|\mathbf{r}\|^2} \cdot \dfrac{\mathrm{d}r_1}{\mathrm{d}\alpha_{12}} - \dfrac{\alpha_{22}}{\|\mathbf{v}_2\|^2} \\ \dfrac{\mathrm{d}\eta_2}{\mathrm{d}z} = -\dfrac{r_2}{\|\mathbf{r}\|^2} \cdot \dfrac{\mathrm{d}r_1}{\mathrm{d}\alpha_{12}} - \dfrac{\alpha_{11}}{\|\mathbf{v}_1\|^2} \cdot \dfrac{\mathrm{d}\alpha_{21}}{\mathrm{d}\alpha_{12}} \end{cases} \tag{S24}$$

Supplementary References

Ted J. Case. *An illustrated guide to theoretical ecology*. Oxford University Press, New York ; Oxford, 2000. ISBN 978-0-19-508512-9.

Dmitriĭ Olegovich Logofet. *Matrices and graphs: stability problems in mathematical ecology*. CRC Press, Boca Raton, 1993. ISBN 978-0-8493-4246-2.

Robert MacArthur and Richard Levins. The limiting similarity, convergence, and divergence of coexisting species. *The american naturalist*, 101(921):377–385, 1967.

John H. Vandermeer. Interspecific Competition: A New Approach to the Classical Theory. *Science*, 188(4185):253–255, April 1975. doi: 10.1126/science.188.4185.253.

Vito Volterra. *Leçons sur la théorie mathématique de la lutte pour la vie*. Gauthier-Villars, Paris, 1931.
